# Identification of carbohydrate gene clusters obtained from in vitro fermentations as predictive biomarkers of prebiotic responses

**DOI:** 10.1186/s12866-024-03344-y

**Published:** 2024-05-25

**Authors:** Car Reen Kok, Devin J. Rose, Juan Cui, Lisa Whisenhunt, Robert Hutkins

**Affiliations:** 1 Complex Biosystems, University of Nebraska-Lincoln, Lincoln, NE 68588 USA; 2 Nebraska Food for Health Center, University of Nebraska-Lincoln, Lincoln, NE 68588 USA; 3 Department of Food Science and Technology, University of Nebraska, 268 Food Innovation Center, Lincoln, NE 68588 USA; 4 Department of Computer Science and Engineering, University of Nebraska-Lincoln, Lincoln, NE 68588 USA; 5 Physical and Life Sciences Directorate, Lawrence Livermore National Laboratory, Livermore, CA 94550 USA; 6 Department of Food Science and Technology, University of Nebraska, 258 Food Innovation Center, Lincoln, NE 68588-6205 USA

**Keywords:** Microbiome, Prebiotics, CAZymes, Personalized nutrition

## Abstract

**Background:**

Prebiotic fibers are non-digestible substrates that modulate the gut microbiome by promoting expansion of microbes having the genetic and physiological potential to utilize those molecules. Although several prebiotic substrates have been consistently shown to provide health benefits in human clinical trials, responder and non-responder phenotypes are often reported. These observations had led to interest in identifying, a priori, prebiotic responders and non-responders as a basis for personalized nutrition. In this study, we conducted in vitro fecal enrichments and applied shotgun metagenomics and machine learning tools to identify microbial gene signatures from adult subjects that could be used to predict prebiotic responders and non-responders.

**Results:**

Using short chain fatty acids as a targeted response, we identified genetic features, consisting of carbohydrate active enzymes, transcription factors and sugar transporters, from metagenomic sequencing of in vitro fermentations for three prebiotic substrates: xylooligosacharides, fructooligosacharides, and inulin. A machine learning approach was then used to select substrate-specific gene signatures as predictive features. These features were found to be predictive for XOS responders with respect to SCFA production in an in vivo trial.

**Conclusions:**

Our results confirm the bifidogenic effect of commonly used prebiotic substrates along with inter-individual microbial responses towards these substrates. We successfully trained classifiers for the prediction of prebiotic responders towards XOS and inulin with robust accuracy (≥ AUC 0.9) and demonstrated its utility in a human feeding trial. Overall, the findings from this study highlight the practical implementation of pre-intervention targeted profiling of individual microbiomes to stratify responders and non-responders.

**Supplementary Information:**

The online version contains supplementary material available at 10.1186/s12866-024-03344-y.

## Introduction

The composition of the human gut microbiome is unique to each individual, highly variable even within similar populations, and resistant to change [1–3]. Thus, in clinical trials, dietary interventions directed at improving health outcomes via modulation of the gut microbiome often result in variable responses. Such studies often lead to individuals who react favourably (i.e., responders) towards the intervention and individuals that either do not present a response or present an unfavourable response (non-responders) [4–6]. These observations suggest that personalized approaches to modulate the gut microbiome in a precise and consistent manner may provide a basis for enhancing responder rates [7, 8].

One such approach is via the consumption of nondigestible oligosaccharides or prebiotic dietary fibers—substrates that enrich host microorganisms that are considered to reduce disease risk and provide benefits to the host [9]. Although numerous studies have shown that prebiotic fibers enrich for bifidobacteria and can contribute to host health, responses are highly individualized [4, 5, 10–12]. Indeed, in several prebiotic trials [6, 13–15], non-responder rates (based on both taxonomic and clinical outcomes) of about 50% have been reported.

In general, two main approaches for developing personalized fiber interventions have been proposed. One way would be to design structurally unique fibers whose metabolism in the gut is precise and targeted [16]. Alternatively, if individual microbiomes were sufficiently interrogated, models can be developed based on microbiome features to predict substrates that are likely to elicit the expected response [17]. Accordingly, several microbiome studies have demonstrated successful selection of predictive biomarkers using machine learning approaches on data collected from hundreds to thousands of individuals [7, 18, 19]. However, given the wide range of prebiotic substrates available and the complexity and individuality of the gut microbiome [20], the ability to predict if an individual is likely to benefit from a particular prebiotic remains challenging.

Several studies have performed biomarker discovery by first stratifying individuals into responder and non-responder groups based on an observed pre-defined diagnostic or clinical response [2, 4, 5, 21]. Then, discriminatory features were identified between groups by comparing gut microbial composition in pre-intervention fecal samples. Most of these studies have implemented a taxonomic approach that, although informative, is limited by its inability to discern accurate functional differences between phenotypes [22–24].

Moreover, recent findings suggest that differential responses are likely driven by specific strains capable of metabolizing different substrates, which cannot be differentiated using a purely taxonomic approach [16, 25, 26]. This is especially relevant for utilization of complex carbohydrates and prebiotic fibers, where carbohydrate active enzymes (CAZymes) of bacterial origin play a major role in carbohydrate catabolism [27]. Specific carbohydrate transporter genes are also required to shuttle these substrates and/or their smaller subunits across cell membranes prior to intracellular metabolism [28–30]. Thus, without the relevant genetic machinery to degrade and metabolize a prebiotic, either through direct microbe-substrate interactions or through cross-feeding reactions, it is highly unlikely that an individual would be able to benefit from its consumption.

In this study, we hypothesized that prebiotic responders share prebiotic-specific carbohydrate degradative systems required for effective utilization. Based on the well-established role of short chain fatty acids (SCFA) as beneficial biomarkers, SCFA production was used here as the targeted response. Accordingly, we used a combination of in vitro fermentations, shotgun metagenomic sequencing, and supervised machine learning to develop predictive tools to identify, a priori, prebiotic response phenotypes. The predictive models were subsequently tested and validated in a human feeding trial across three well-studied prebiotics, xylooligosaccharides (XOS), fructoligosaccharides (FOS), and inulin.

## Results

### SCFA profiles are dependent on prebiotic type and are individualized

**Fig. 1:**
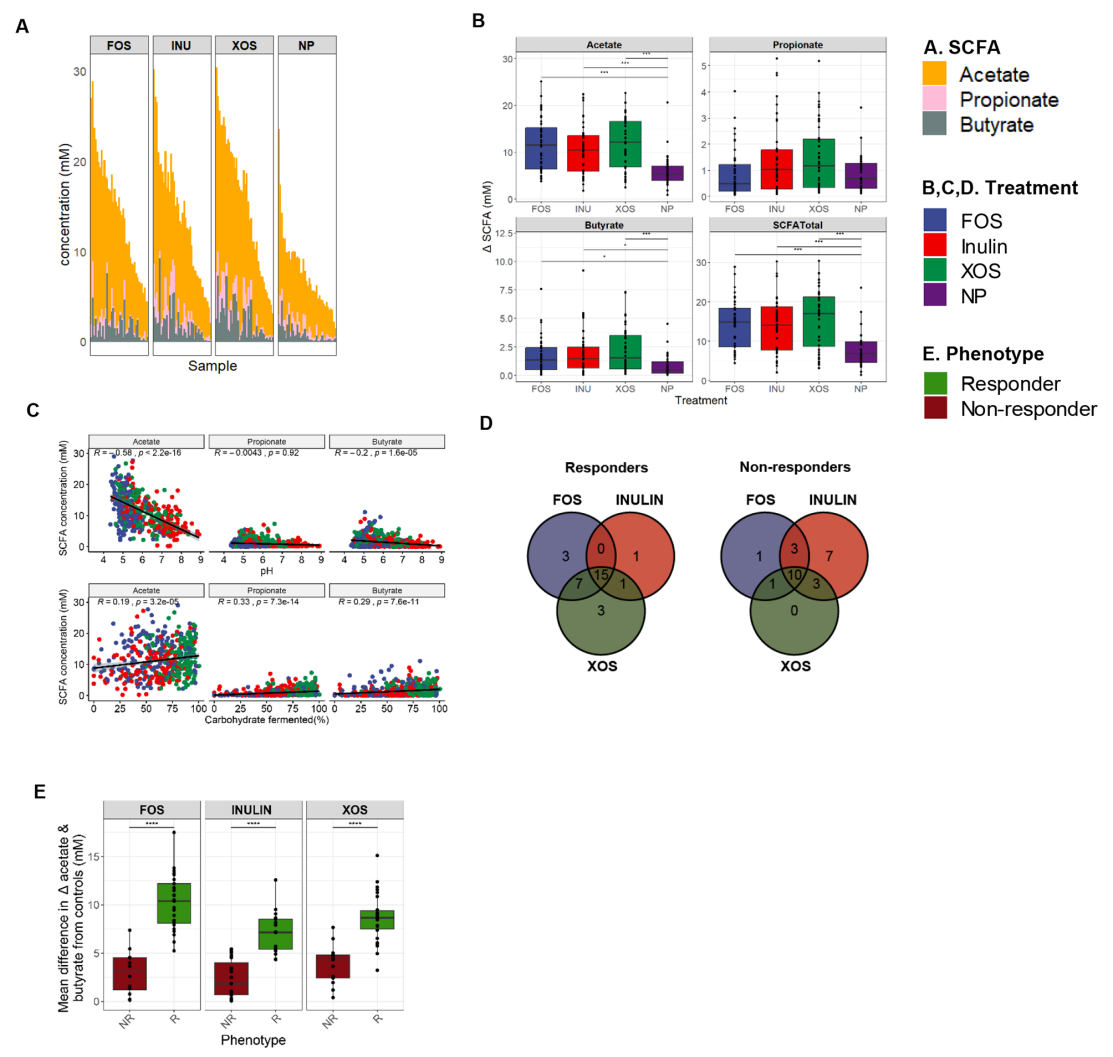
SCFA production of in vitro fecal fermentations and classification of prebiotic responders and non-responders. (**A**) Average concentrations of acetate, butyrate, and propionate across four timepoints from 40 fermentations. Samples are arranged according to decreasing sums of acetate and butyrate concentrations. (**B**) Differences in SCFA production between treatments (ANOVA followed by pairwise t-test; Holm-Bonferroni adjusted *, *p* < 0.05; **, *p* < 0.01; ***, *p* < 0.001). Baseline concentrations were subtracted from the measured SCFA concentrations. (**C**) Correlations between individual SCFA with pH and the percentage of fermented carbohydrate. Each data point represents a sample collected after every 12-hour cycle per subject and is an average of duplicate experiments. The shaded regions represent 95% confidence intervals. Colors represent the treatments; FOS: blue, inulin; red, XOS: green, NP (no prebiotic added as a control): purple. (**D**) Venn diagram depicting the number of shared responders (R) and non-responders (NR) across each prebiotic substrate. (**E**) Mean difference in acetate and butyrate concentrations between prebiotic-treated samples compared to no-prebiotic controls across responders and non-responders (t-test, ****, *p* < 0.0001). Each data point corresponds to the average value across timepoints for a given fermentation. Baseline concentrations were subtracted from the reported sum of SCFA concentrations. Colors correspond to each phenotype; responders: green and non-responders: red. The upper and lower hinges of boxplots correspond to the 75% and 25% quantile respectively. The upper whiskers correspond to the largest observation less than ‘*75th percentile + 1.5 X interquartile range’* while the lower whiskers correspond to the smallest observation greater than ‘*25th percentile – 1.5 X interquartile range’*

A stepwise in vitro fermentation approach was used to identify responder and non-responder phenotypes based on SCFA production. Accordingly, fermentations were started by adding fecals slurries to fermentation broth containing one of three prebiotics (FOS, inulin, and XOS). Dilution pressure was maintained by making 100-fold dilutions every 12 h into fresh fermentation broth with fresh fecal spike-ins. Fermentations were performed using fecal samples obtained from 40 individuals, in duplicate, and SCFA concentrations were quantified for every subject-prebiotic combination across four timepoints. As no significant interactions were observed over time, SCFA means across time points were used to phenotype subjects either as responders or non-responders. Accordingly, acetate made up the highest proportion of total SCFA, followed by butyrate and propionate (Fig. 1A). Overall, concentrations of acetate, butyrate and total SCFA were significantly higher for all three substrates compared to the no-prebiotic controls (Fig. 1B; Holm-Bonferroni adjusted *p* < 0.05), However, no significant differences in propionate concentrations were observed.

Fermentations were also evaluated by relating SCFA concentrations to pH and carbohydrate consumption. Although most of the terminal pH values ranged between 4.5 and 7.5, some samples reached as high as pH 9.0, presumably because alkaline end-produced were produced from amino acids by non-responder microbiotas. Acetate and butyrate concentrations were inversely correlated with pH and positively correlated with carbohydrate consumption while propionate correlated positively with the percent of fermented carbohydrate (Fig. 1C). Thus, concurrent with the high and stable production of acetate and butyrate across all prebiotic treatments, and significant correlations with other fermentation parameters, the sum of acetate and butyrate concentrations were used as a reliable read-out to assess prebiotic response in the in vitro system.

Acetate and butyrate concentrations were compared between every prebiotic treatment with parallel negative controls to assign phenotypes to individual samples (Fig. S1). Accordingly, based on the SCFA concentration differences between the treatment and control fermentations,15 subjects were classified as responders, and 10 subjects were non-responders across all three prebiotics. Seven subjects were responders to only one substrate and eight subjects were responders towards two substrates (Fig. 1D**).** On a prebiotic basis, there were 26 XOS responders (65.0%), 25 FOS responders (62.5%) and 17 inulin responders (42.5%), with an average response rate of 57.0%. Also, all responders had a significantly higher mean difference in acetate and butyrate concentrations from controls, compared to non-responders (Fig. 1E). Overall, these results demonstrate that SCFA profiles varied in response towards both the type of prebiotic and the donor fecal sample which could be informative in discerning between responders and non-responders.

### Shotgun metagenomic sequencing reveals distinct response-dependent taxonomic and CAZyme profiles

**Fig. 2:**
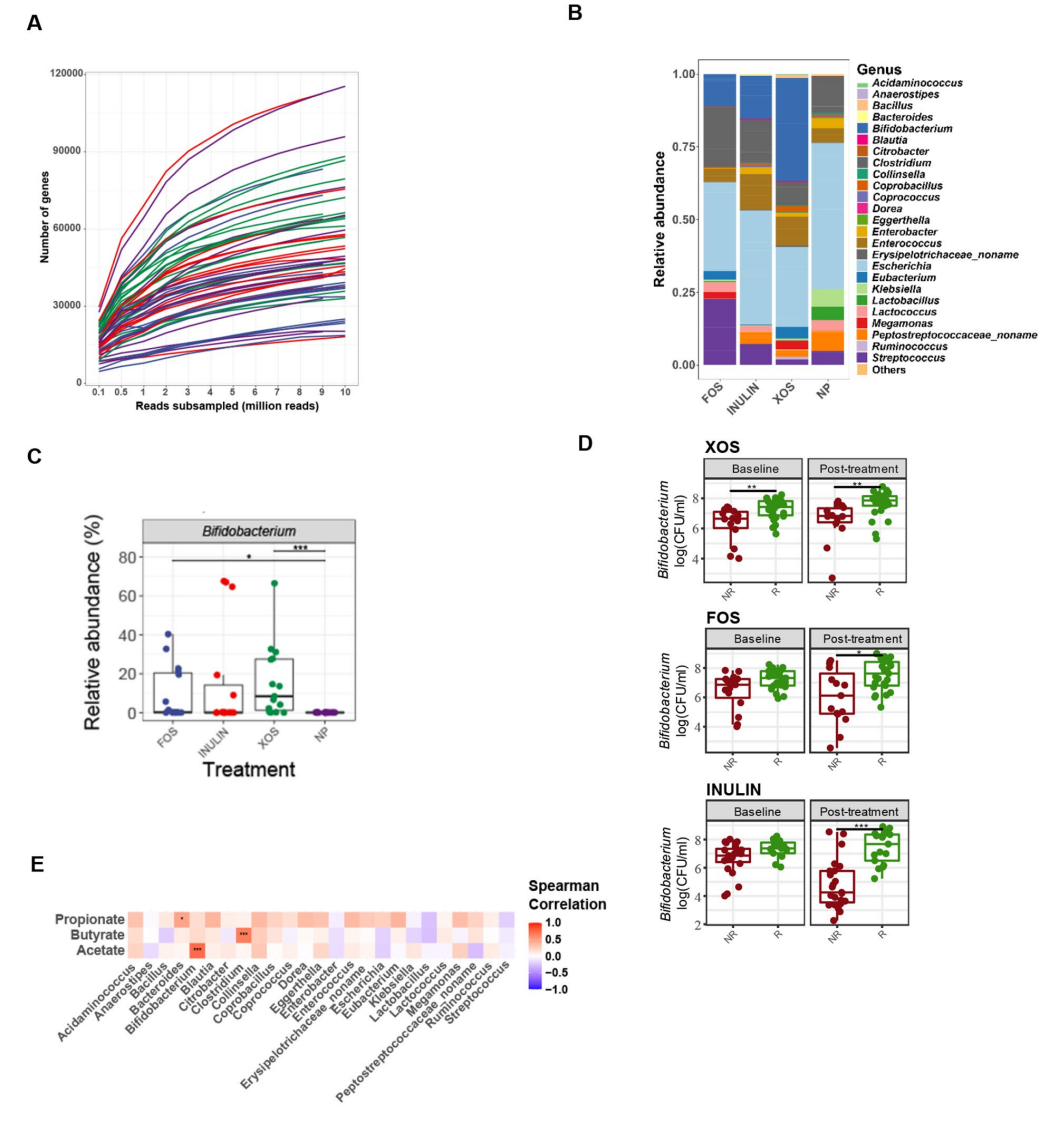
Shotgun metagenomic sequencing of post-fermentation samples and bifidogenic response. (**A**) Rarefaction curve displaying the effect of sequencing depth on the number of new genes. Each line represents a unique metagenome sample (*n* = 15). (**B**) Mean relative abundance of taxonomic species at the genus level across samples for each prebiotic treatment. Taxonomic species that are present at less than 0.1% relative abundance are grouped as “Others”. (**C**) Significant differences in relative abundances of *Bifidobacterium* between prebiotic treatments. Each data point represents a metagenome sample (*n* = 15). (**D**) Significant differences in *Bifidobacterium* counts between responders (R) and non-responders (NR) for each prebiotic as determine through qPCR (*n* = 40). Wilcoxon tests were used to determine significant differences in taxa (FDR adjusted *, *p* < 0.05; **, *p* < 0.01; **, *p* < 0.001). (**E**) Correlations between SCFA production with genus relative abundances (FDR adjusted *, *p* < 0.05; **, *p* < 0.01; ***, *p* < 0.001)

Shotgun metagenomic sequencing was performed on a subsample of fifteen subjects across four treatments after the 48 h fermentation timepoint (*n* = 15 per treatment). Although not completely converged, the asymptotic shape of the rarefaction curves suggests that the sequencing depth was sufficient for identifying abundant genes or species (Fig. 2A). Pairwise Wilcoxon tests were used to determine significant differences between treatments across taxa at the genus and species level. At the genus level, *Bifidobacterium* abundance was observed to be significantly higher in XOS and FOS fermentations compared to the no prebiotic control (FDR adjusted *p* < 0.05, Fig. 2B, C). Other genera that appeared at high abundance in both the prebiotic and no prebiotic treatments included *Escherichia*, *Citrobacter*, and *Streptococcus.* No significant differences were found between treatments at the species-level.

To further assess the bifidogenic effect of these substrates, qPCR quantification was used to compare absolute cell numbers of *Bifidobacterium* between responders and non-responders. The results showed that there were significantly higher *Bifidobacterium* counts in responders compared to non-responders across all three substrates after fermentation (Fig. 2D). At baseline, *Bifidobacterium* counts were significantly lower in XOS non-responders compared to responders. Correlation analyses between genus abundance and SCFA concentrations showed positive correlations between *Bifidobacterium* with acetate (r 0.73, adjusted *p*-value < 4.4e-8) and *Clostridium* with butyrate (r 0.65, adjusted *p*-value < 6.2e-6) (Fig. 2E). Propionate, in contrast, was positively correlated with *Bacteroides* (r 0.47, adjusted *p*-value 0.011), although average propionate concentrations were relatively low (< 1 mM) across all subjects.

**Fig. 3:**
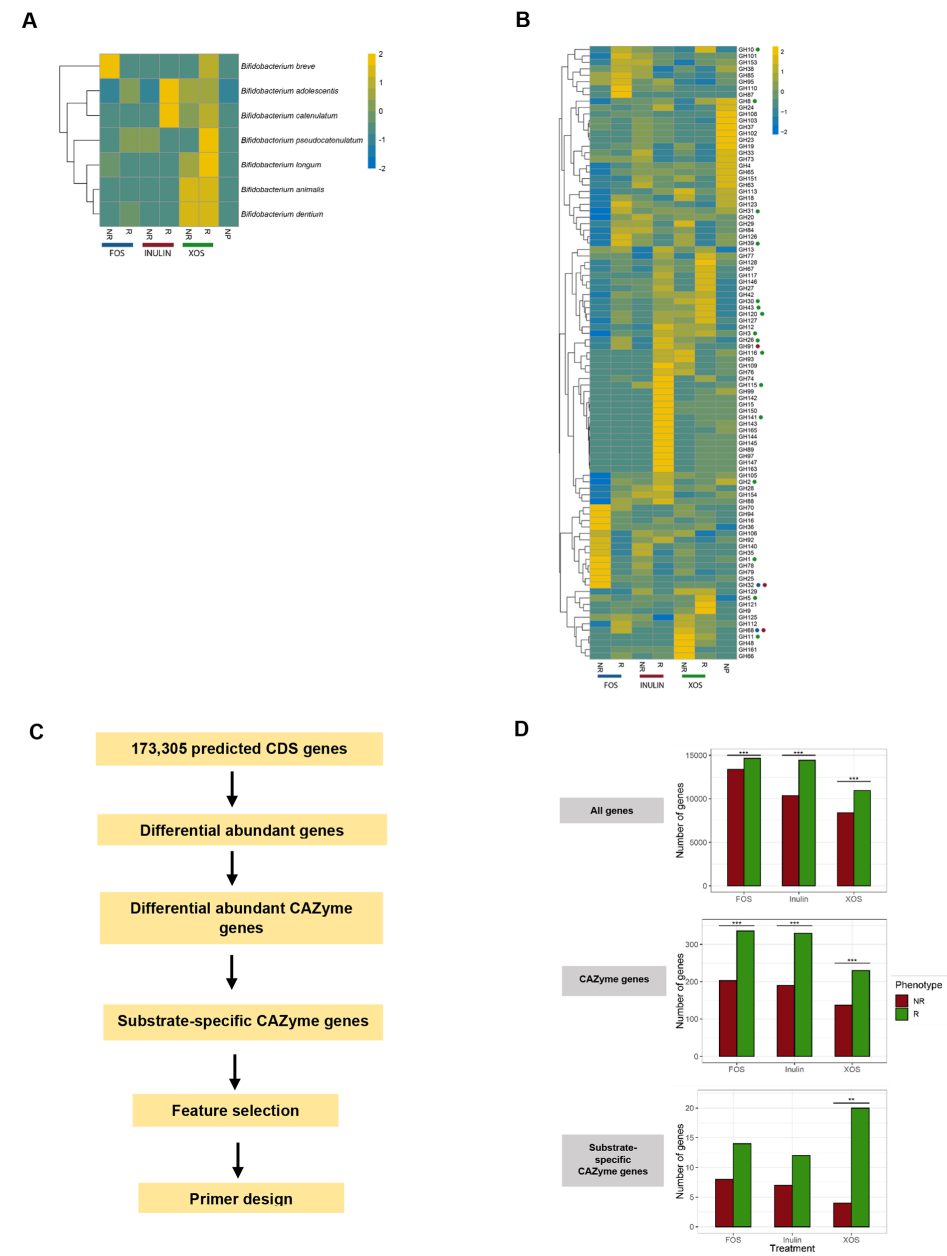
Targeted selection of candidate prebiotic-associated CAZyme genes identified from fecal metagenomes. **A**, **B**) Heatmaps visualizing the hierarchical clustering of *Bifidobacterium* species (**A**) and CAZyme families (**B**) based on z-score normalization of mean relative abundances obtained from metagenomes. Each column corresponds to responder (R) and non-responder (NR) phenotypes across prebiotic treatments. CAZymes that are associated with FOS, inulin and XOS based on annotations from the CAZy database are annotated with blue, purple and green circles, respectively. Abundances of taxonomic species and GH families were standardized across rows and clustered hierarchically according to similar patterns of abundance. **C**) Flowchart of CAZyme gene discovery and annotation. Differential abundant genes were assigned as CAZymes using dbCAN2 and substrate specific genes were defined according to CAZyme family annotations that were relevant to the prebiotic of interest. Responder-associated substrate specific genes were selected for feature selection. **D**) The number of genes associated with responders (R) and non-responders (NR) per substrate are visualized using bar charts after each filtering step. Chi-square goodness of fit tests were used to determine significant differences in the number of differential abundant genes between responders and non-responders across each prebiotic treatment (Holm-Bonferroni adjusted **, *p* < 0.01; **, *p* < 0.001)

Species-level profiles across treatments were visualized using hierarchical clustering of relative abundances (Fig. 3A, Fig. S2**A**). *Bifidobacterium* species in XOS responders were mainly comprised of *Bifidobacterium longum* (16.06%), *Bifidobacterium pseudocatenulatum* (14.54%), and *Bifidobacterium adolescentis* (13.17%), whereas for FOS and inulin responders, *Bifidobacterium adolescentis* was the major *Bifidobacterium* species (FOS; 13.05%, inulin; 25.33%) (Fig. S3). These findings suggest that *B. adolescentis* strains might have functionally distinct roles depending on the substrate. In addition, the high percentage of *Clostridium* species in the fructan responder samples consisted mainly of *Clostridium perfringens* (FOS: 29.17%, inulin: 5.04%), followed by *Clostridium butyricum* (FOS: 1.53%, inulin: 3.48%). *C. perfringens* also made up a high proportion of *Clostridium* species in inulin non-responder samples (18.17%). Additionally, *Streptococcus* species appeared to be abundant in all non-responder samples and were mostly comprised of *Streptococcus pasteurianus* (FOS: 34.36%, inulin: 14.07%, XOS: 4.61%) and *Streptococcus lutetiensis* (FOS: 19.03%, inulin: 0.25%, XOS: 0.09%).

An overall enrichment of genes associated with Clusters of Orthologous Groups (COG) annotation of carbohydrate transport and metabolism was observed in prebiotic fermentations compared to no-prebiotic controls (Fig. S2**B**). Thus, to investigate genes responsible for saccharolytic activity in these fermentations, we utilized dbCAN2 to predict and assign genes into CAZyme families. Hierarchical clustering of GH families across samples provided a visual representation of the abundance and distribution across prebiotic treatments and phenotypes (Fig. 3B). In XOS responders, xylan associated CAZyme families GH43 and GH120 were clustered together. In inulin responders, however, GH91 (inulinase) clustered with other GH families such as GH26 (β-mannanase), GH89 (α-N-acetylglucosaminidase), GH150 (I-carrageenase), and GH165 (β-galactosidase), all of which are not relevant for inulin metabolism according to current annotations. This observation is likely a result of an increase in a particular bacterial strain or species that also harbours these genes. We further observed that the fructan-associated CAZyme family, GH32, was not abundant in FOS and inulin responders and instead was prevalent in FOS non-responder samples. This is likely attributed to the size of the GH32 family that also includes sucrose-6-phosphate hydrolase, an enzyme commonly involved in sucrose metabolism and reported to be present across many taxa.

### Discovery and characterization of prebiotic-specific carbohydrate genes and carbohydrate gene clusters (CGC)

Differential analysis for all 173,305 predicted genes was conducted to identify differentially abundant (DA) genes between responders and non-responders for each prebiotic treatment (Fig. 3C, D). The number of DA genes and DA CAZyme-encoding genes in responders were higher compared to non-responders across all treatments (Fig. 3D). Using existing annotations in the CAZy database, we first investigated specific GH families and subfamilies involved in xylan and fructan degradation as listed in Table S2 to obtain a list of substrate-specific CAZyme genes. These included GH32 and GH68 for FOS and GH32, GH68 and GH91 for inulin. CAZyme families involved in XOS degradation included GH8, GH10, GH43, GH120, among many others. The number of DA substrate specific CAZyme genes were significantly higher in responders for XOS.

Genes were further annotated and assigned EC numbers with eggNOG-mapper and their respective functions (Table S3). The CAZyme families for FOS and inulin have relatively fewer subfamilies and EC numbers with most genes denoted as EC 3.2.1.26, corresponding to β-fructofuranosidase. For FOS fermentations, thirteen GH32 genes were significantly higher in responders and nine were significantly higher in non-responders. In inulin fermentations, two GH91 and ten GH32 genes had a significantly higher fold change in responders and seven GH32 genes were significantly higher in non-responders. Two *Bifidobacterium* GH32-encoding genes; GID_21721 and GID_93934 were significantly enriched in both FOS and inulin responders, implying shared roles in degradation of both fructans. In addition, multiple *S. pasteurianus* GH32-encoding genes were enriched in fructan non-responders. All FOS and inulin GH32-encoding genes that had an increase in fold-change were retained as substrate-specific responder-associated genes.

For XOS fermentations, EC assignments revealed that many genes were irrelevant to XOS metabolism. For example, most of the GH1 and GH3-assigned genes were β-glucosidases while most of the GH2-assigned genes were β-galactosidases. Other relevant significantly enriched genes in responders included one GH8, one GH120 and thirteen GH43 encoding-genes, while in non-responders, four GH43 encoding-genes were significantly higher. Overall, the number of these prebiotic-specific glycosyl hydrolases were higher in responders compared to non-responders across all prebiotics and were mostly derived from *Bifidobacterium*. Based on CAZyme assignments and EC number annotations, genes that were annotated as β-glucosidase, β-galactosidase, β-manosidase, β-N-acetylhexosaminidase and arabinan endo-1,5 L-arabinosidase were removed and a list of 38 substrate-specific responder-associated genes was retained (Table S4).

### Feature selection and primer design

**Fig. 4:**
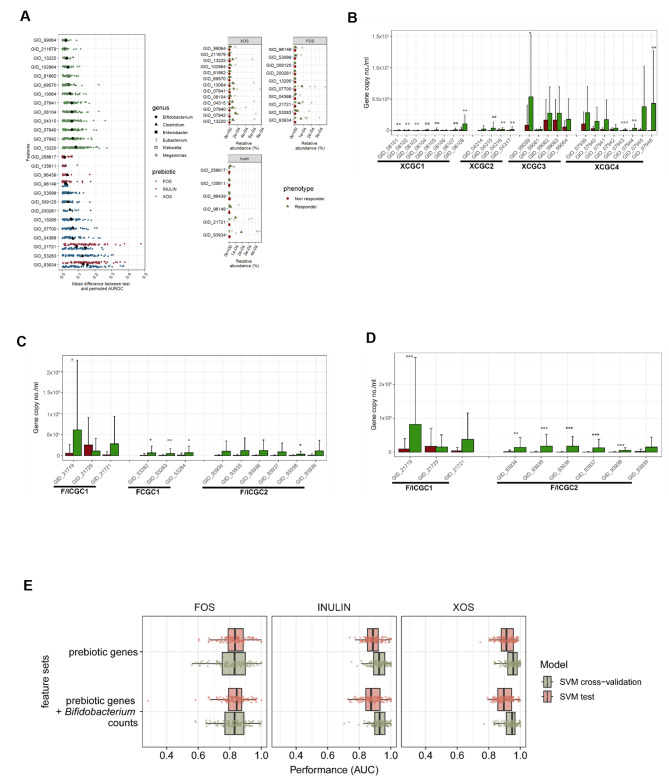
Feature selection and quantification of prebiotic responder-associated genes for the prediction of prebiotic responder and non-responder phenotypes. **A**) Feature importance comprising of CAZyme-encoding genes enriched in prebiotic responders as determined through feature permutation of SVM models. Features are ranked based on mean differences between test and permuted area under the curve (AUC) across 100 different models. Each data point represents a calculated difference with colors representing a prebiotic substrate. Data points in black indicates the mean difference with symbols representing taxa. The relative abundance of each gene is also shown here across all three prebiotics. **B**, **C**,**D**) qPCR quantification of *Bifidobacterium* and prebiotic responder-associated genes at baseline for XOS (**B**), FOS (**C**) and inulin (**D**) fermentations. Wilcoxon tests were used to determine significant differences between responders and non-responders for each gene (Holm-Bonferroni adjusted *, *p* < 0.05; **, *p* < 0.01; ***, *p* < 0.001). Colors correspond to responders (green) and non-responder (red) phenotypes. **E**) Support vector machine (SVM) models with the Radial Basis Function (RBF) kernel were built using either baseline prebiotic genes or a combination of prebiotic genes and *Bifidobacterium* counts as features. The vertical lines in each boxplot represents the median AUC across 100 random permutations per prebiotic

Feature permutation across 100 Support Vector Machine (SVM) models with the Radial Basis Function (RBF) kernel was used to rank discriminatory features according to order of importance prior to selection of targeted genes for absolute quantification (Fig. 4A). Genes belonging to *Bifidobacterium* were ranked highest across all substrates with relatively higher abundance in responders compared to non-responders. Lower ranking features, however, do not indicate that the genes were irrelevant. Instead, this could be attributed by their limited presence in only a few responder subjects.

We also investigated neighbouring accessory genes to assess their role as responder-associated genes for carbohydrate uptake and metabolism in a context-dependent manner. These carbohydrate gene clusters (CGC) were identified using the CGCFinder functionality in dbCAN2 and are comprised of CAZyme-genes that are linked to sugar transporter genes and other regulatory elements (Table S5). Some gene clusters contain several responder-associated genes. Among the types of transporters identified were ATP-binding cassette (ABC) transporters, major facilitator superfamily (MFS) transporters, and phosphotransferase systems (PTS).

Based on the feature permutation results and the overall bifidogenic responses taxonomically and functionally, the top-ranking *Bifidobacterium*-derived genes were selected as predictive features to distinguish responders from non-responders. As carbohydrate uptake and metabolism require intact genetic machinery to be present, genes within entire gene clusters were considered for downstream predictive analyses. In total, four XOS (XCGC1, XCGC2, XCGC3 and XCGC4) and three fructan (FCGC1 for FOS; and F/ICGC1 and F/ICGC2 for both FOS and inulin) responder associated CGC were selected. Unique CGC identifiers were assigned to each cluster according to the substrate that it is associated with (“X”, “F” or “I”), followed by a number (Table S5). Further inspection of these gene clusters revealed that XCGC1, XCGC2, XCGC3 and XCGC4 (CGCs for XOS) harbored β-xylosidase encoding genes. XCGC4, in particular, contained 5 ABC transporters and 5 CAZymes, including one GH8 gene and one GH120 gene. The clusters that included GID_21721 and GID_93934 were present in both FOS and inulin responders and were referred to as F/ICGC1 and F/ICGC2, respectively.

### Carbohydrate genes are detected at baseline and are discriminatory

After identifying the relevant genes and CGC upstream, primers were designed to target each gene in all seven gene clusters (37 genes; Table S6). The copy number of the selected genes in pre-fermentation baseline samples of all 40 individuals were determined by qPCR. The results demonstrated that the targeted genes were detectable at baseline (Fig. 4. **B, C,D**). The estimated copy number of each gene was subsequently compared between responders and non-responders. Significantly different genes between phenotypes were identified within all targeted prebiotic-associated clusters. Interestingly, only the transporter genes comprising ABC substrate binding proteins and permeases in XCGC4 (GID_07943, GID_07944, and GID_07946) were significantly different between both phenotypes (Fig. 4B). Similarly, the ABC substrate binding protein, GID_21719 was the only gene in F/ICGC1 that was significantly different between phenotypes in both FOS and inulin treatments (Fig. 4C, D).

As noted previously, F/ICGC1 and F/ICGC2 were identified as responder-associated gene clusters in both FOS and inulin treatments. However, although the copy number of all genes in FCGC1 were significantly higher in FOS responders compared to non-responders at baseline, this gene cluster was not discriminatory between inulin responders and non-responders. Conversely, most of the F/ICGC2 genes (5 out of 6) were significantly higher in inulin responders (Fig. 4D) compared to non-responders, while only GID_93938 was significantly different for FOS (Fig. 4C). These results imply that each of these gene clusters have specialized roles in uptake and degradation of fructans of differing complexity and degree of polymerization.

### Support vector machine classifiers for prediction of response phenotypes

The selected features were used to train RBF SVM classifiers to classify responders and non-responders for each prebiotic. There were twelve, nine, and twenty-four genes associated with FOS, inulin and XOS response, respectively. For each substrate, SVM models were built using features that consisted of either *Bifidobacterium* prebiotic-associated gene copy number only or combined with *Bifidobacterium* counts. Model accuracy was measured according to the area under the curve (AUC) (Table S7). According to the AUC reported for the models using *Bifidobacterium* prebiotic-associated genes only, the XOS model achieved the highest classification accuracy (train: 0.95, test: 0.91), followed by inulin (train: 0.93, test: 0.90) and FOS (train: 0.82, test: 0.83) (Fig. 4E). In addition, the combined use of *Bifidobacterium* counts and *Bifidobacterium* prebiotic genes either lowered (XOS, inulin) or did not alter (FOS) model accuracy. These results suggests that gene features identified from the metagenomes of fecal fermentations were predictive of prebiotic response phenotypes in vitro, with high accuracy for XOS and inulin treatments.

### Xylooligosaccharide model predicts SCFA response in vivo

**Fig. 5:**
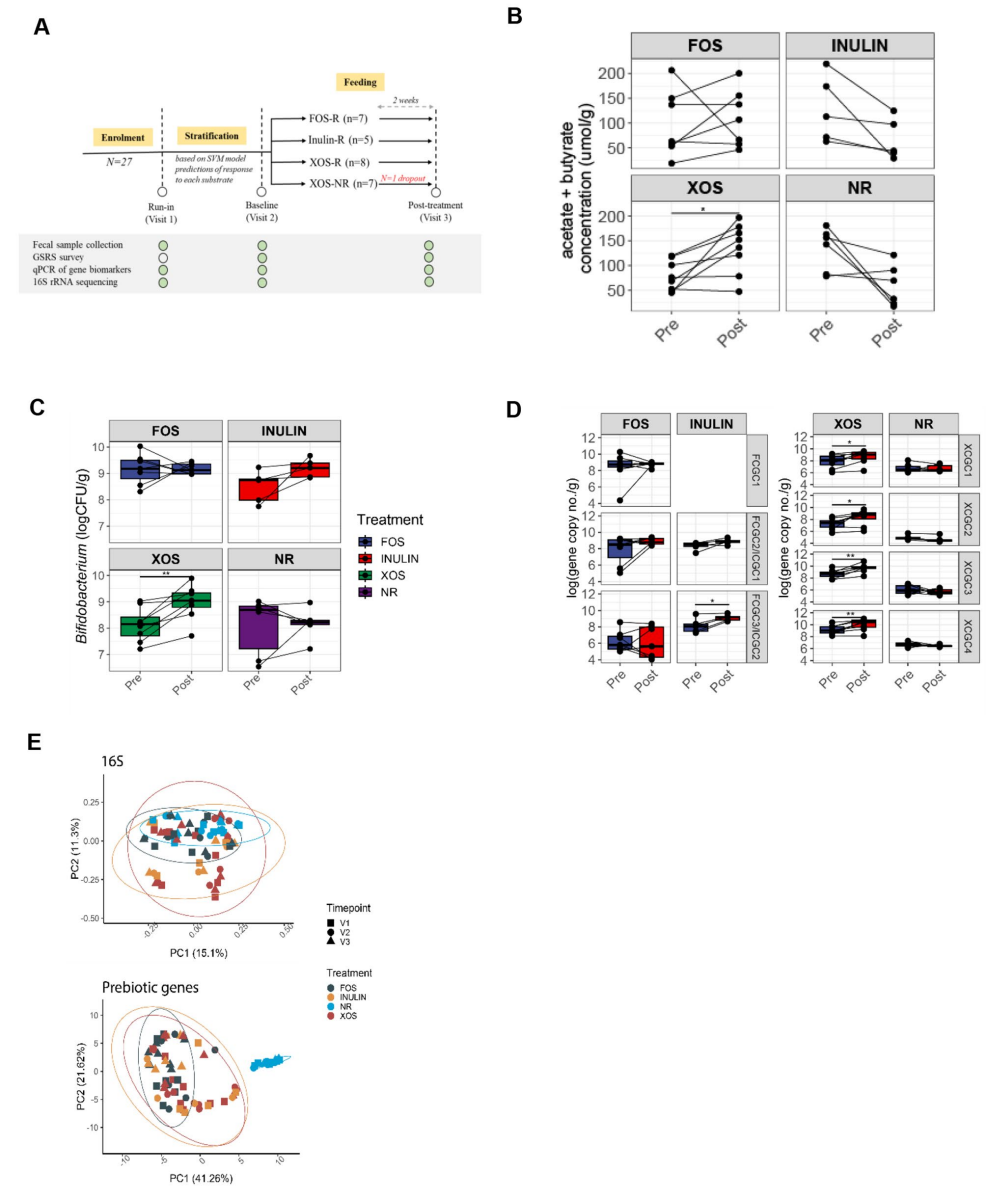
Prediction and stratification of subjects as prebiotic responders and non-responders in a human feeding study. (**A**) A feeding trial was conducted using a single-blind multi-arm parallel-group stratified design whereby subjects were stratified to different treatment groups according to their prebiotic-associated gene profiles. Subjects were given prebiotics to consumed for a 2-week period. Green circles represent data that was collected per visit. **B**, **C**,**D**) Changes in the sum of acetate and butyrate (**B**) *Bifidobacterium* counts (**C**) and prebiotic-associated gene copy numbers (**D**) before and after the feeding period (paired t-test; *, *p* < 0.05; **, *p* < 0.01). Horizontal lines connect data points from samples of the same individual. **E**) Beta diversity ordination plots (PCoA) of Bray-Curtis dissimilarity matrix using either 16S taxonomic profiles or prebiotic-associated gene profiles as features

To test the model in vivo, a blinded pilot clinical feeding trial was conducted in healthy human subjects (*n* = 27). During run-in, fecal samples were collected and prebiotic prediction features were assayed and used to predict responders and non-responders (Fig. 5A). Prebiotics were administered according to their predicted phenotypes, resulting in seven predicted XOS responders, seven FOS responders, and five inulin responders. All participants received the assigned prebiotic (5 g per day) for a period of two weeks. Eight subjects were predicted to be non-responders for all prebiotics and were given XOS and served as a non-responder control group. No significant differences in gastrointestinal symptoms were reported before and after intervention for any of the treatment groups (Table S8). However, three subjects reported flatulence scores of 5 and 7 at visit 3 towards inulin and XOS, respectively (Table S9). Similarly, there were no significant associations found between reported dietary habits and response phenotypes (Table S10, S11, S12).

Following the in vitro experiments, we evaluated changes in the sum of acetate and butyrate in fecal samples pre- and post-prebiotic intervention to confirm predicted response phenotypes (Fig. 5B). Using a two-way repeated measure ANOVA, significant increases were observed in the XOS-responder group, primarily in acetate while significant shifts in SCFA were not observed in other treatment groups (Fig. S4; *p* < 0.05). Therefore, overall responder phenotypes were not reflected, in vivo, by increased SCFA production, as observed in the in vitro fermentations. Taxonomic changes were also observed, specifically a consistent increase in *Bifidobacterium* abundance in all subjects within the XOS groups (Fig. 5C, Fig. S4B). In contrast, the FOS responder and XOS non-responder control group displayed varied and individualized changes in *Bifidobacterium* abundance. In addition, the abundance of targeted gene clusters was evaluated across samples by summation of the gene copy number of each gene within a cluster. A significant increase in prebiotic-associated genes in F/ICGC2 and all XCGC clusters was also observed in the inulin and XOS responder groups respectively (Fig. 5D).

Shifts in microbial diversity between visits were also evaluated. No significant changes in alpha diversity (Shannon index) were observed between visits or treatments at the end of the intervention period **(Fig. S5A, B**). Beta diversity ordination plots (PCoA) of Bray-Curtis dissimilarity matrix demonstrated that the fecal microbiome composition of the same subjects clustered tightly through the three timepoints, but separately from other subjects, reflecting highly individualized composition of the gut microbiome (Fig. S5C-F). Similar ordinations using taxonomic information derived from 16S rRNA sequencing did not reflect differences between responders and non-responders (Fig. 5D). Conversely, ordinations with the prebiotic-associated genes as features revealed a distinct non-responder cluster consisting of samples that did not harbor any targeted prebiotic-associated genes.

## Discussion

Although many mechanistic studies and clinical trials have demonstrated that fiber-rich diets consistently influence the composition and function of the gut microbiome [31–33], these dietary treatments often result in observed changes in only a subset of individuals [4, 6, 34] Indeed, the occurrence of non-responders in dietary clinical trials is very common [35–38]. Thus, a major challenge for researchers and clinicians has been to predict treatment responses, a priori, providing opportunities for practical precision nutrition recommendations [39].

In general, the first step in developing models capable of predicting responder and non-responder phenotypes is to identify clinically relevant and measurable responses to a treatment, based on either previously established or expected outcomes. These measured responses usually include changes in taxonomy, SCFA concentrations, or physiological changes such as body mass index, insulin resistance, or immune biomarkers [5, 6]. Accordingly, in this study, SCFA concentrations from in vitro fecal fermentations were selected as metabolic responses, in part because they are well-known to have an important role in gastrointestinal health [40] and also because they serve as consistent, discriminatory, and relevant measures of prebiotic utilization [34]. Acetate and butyrate concentrations, in particular, were used to distinguish responders from non-responders. For many individuals, similar responses were observed regardless of the prebiotic substrates used, implying shared involvement of microbial communities in the utilization of these common prebiotics. On average, the in vitro responder rate per prebiotic was 57%, consistent with several human prebiotic feeding studies, where responder rates of around 50% (based on taxa or functional outcomes) have been observed [5, 12, 13, 41].

Although acetate and butyrate concentrations were reliable response markers in vitro, the in vivo measurements of these SCFA from the feeding trial were generally less informative. Instead, increases in *Bifidobacterium* abundance and specific gene clusters were observed across individuals in the XOS and inulin responder groups. This might be due to unreliable quantification of SCFA directly from fecal samples in vivo as SCFA are rapidly absorbed by colonocytes after production in the colon [42, 43]. Additionally, the 5 g per day doses may have been insufficient to elicit significant differences in fecal SCFA concentrations. Improvements in measuring SCFA flux in vivo, such as through breath and serum samples, may be necessary for model testing [44–47]. In addition, an ex-vivo approach using short timescales has been proposed to evaluate potential SCFA production directly from fecal samples [48].

Ultimately, our goal was to identify features prior to a fiber intervention that could predict an individual’s ability to metabolize that substrate. Glycosyl linkages are abundant among prebiotic structures and their degradation requires CAZymes such as glycosyl hydrolases (GH) [27, 49]. Therefore, to identify potential gene targets for screening prebiotic responders, we selected GH families based on annotations that were specified in the CAZy database. Previous reports on the involvement of some of these GH families (both *in silico* and experimentally) in dietary glycan degradation provided increased confidence for the selection of these GH families [50–54]. In particular, the role of GH32 (includes β-fructofuranosidase and inulinase) and GH91 (includes inulin lyases) in fructan utilization is well established [29, 55–57], while the GH families involved in xylan utilization systems are much more diverse and include GH120 (β-xylosidase), GH8 (includes xylanase), GH43 (includes β-xylosidase) and GH10 (includes xylanase), among many others [58–60]. Given the vast diversity of substrates available, finer CAZyme subfamily resolutions will be necessary to aid the discovery of other prebiotic associated genes to allow for a greater scale of precision. Improvements in CAZyme annotation and curation frameworks as described in Cohen & Borenstein [61]will be especially beneficial in capturing fiber degradative profiles and subsequent biomarker discovery across fiber intervention studies.

Despite evidence of functional redundancy in the gut microbiome [62, 63], shifts in CAZyme composition have previously been observed in response to different substrate feeding [64, 65]. Likewise, in population-based studies, CAZyme profiles have also been shown to reflect dietary preferences and the type of glycans that microbes encounter in the gut [61, 66, 67]. Moreover, strain heterogeneity in CAZyme profiles within the same genus has been described such as in species of *Bifidobacterium* and *Prevotella* [68, 69]. This strain-specific genotype is likely a result of adaptation towards different spatial niches, resulting in the evolution of specialized glycan preferences [70]. Together with horizontal gene transfer events, these factors have led to a high repertoire of glycan degradation systems within the gut that likely translates into the observed inter-individual differences and subsequent responses towards dietary interventions [23, 71]. In our study, the association of unique CGCs towards each fructan indicates the presence of genes specialized towards similar substrates of varying complexity.

In addition to CAZymes, an array of accessory carbohydrate utilization proteins is also necessary for metabolism of prebiotic fibers. As described in Leth et al., (2018) [58], the capture and degradation of different xylan compounds are reflected by the expression of different substrate binding modules and transport systems in gut commensals. Indeed, we observed that genes encoding substrate binding proteins and permeases appeared to be more predictive of responder phenotypes compared to the CAZymes present in the same cluster. This was shown for XCGC4 and F/ICGC1 and implies that the transporter genes in these clusters are essential for substrate uptake and subsequent metabolism. In addition, secretory signal peptides were predicted to be present on the N-terminus of the GID_07946 (XCGC4) permease and GID_21719 (F/ICGC1) substrate binding protein genes suggesting extracellular uptake of substrate. Similarly, Yoshida et al. (2021) [33] demonstrated that the abundance of a solute binding protein was indicative of a bifidogenic response towards lactulose in humans.

Recently, gene clusters homologous to XCGC3 and XCGC4 were reported to be involved in metabolism of arabinoxylan-derived oligosaccharides in *B. pseudocatenulatum* YIT 4072 [59]. The authors experimentally validated the presence of GH43 xylosidases and arabinofuranosidases in both gene clusters [59]. This co-localization (also observed here in XCGC1, XCGC3 and XCGC4) suggests a possible evolutionary adaptation towards arabinoxylan, a major dietary fiber found in cereal grains [72]. The putative role of clusters homologous to F/ICGC2 in FOS and inulin metabolism have also been previously described [51, 73].

Importantly, quantification of baseline *Bifidobacterium* abundance and its inclusion in our models suggest that the abundance of targeted species at baseline is not sufficient for response prediction. Indeed, when abundance of bifidobacteria was included, model accuracy either remained unchanged or decreased slightly. Thus, our in vivo data further emphasizes the potential use of prebiotic gene profiles instead of taxonomic profiles as a stable guide for prebiotic stratification given that taxonomic composition and diversity was highly resilient and individualized over time. Overall, our findings confirm the importance of carbohydrate-associated genes in prebiotic utilization and supports the potential of utilizing a gene profiling approach to differentiate between prebiotic responders and non-responders.

Variation in model performance was observed in vitro with the FOS model performing the worst. Accordingly, the models were able to reliably predict XOS responders both in vitro and in vivo but was less informative in predicting FOS responders. This might be attributed to the accessibility of the short-chain fructan (DP < 10) to a wider range of bacterial species [74] as observed by the enrichment of *Streptococcus* and the corresponding GH32-encoding genes in FOS non-responder samples in vitro. This suggests that robust biomarker selection from the in vitro system could be dependent on substrate complexity and accessibility. Interrogation of a larger number of microbiomes and inclusion of a larger set of predictive features such as other FOS scavenging enzymes is likely required to overcome this and improve model performance. In addition, other non-binary methods for building effective predictive models should also be explored. For example, discretization can be carried out by binning responses into discrete classes (i.e. low, medium, and high responders) while minimizing information loss occurred through binary classification. Furthermore, the use of regression models such as Support Vector Regression (SVR), allowing for the incorporation of quantified responses, should also be considered.

To our knowledge, this study is the first to report the development of predictive models based on genes involved in carbohydrate metabolism beyond a taxonomic approach for making prebiotic recommendations. Overall, our results demonstrate the expansion of *Bifidobacterium*, their saccharolytic genes, and their diverse transport machinery in prebiotic responders, reflecting the evolutionary adaptation of this species for glycan degradation in the gut. Importantly, our findings revealed that the functional composition of baseline microbiomes provides a rational and reliable basis for predicting microbiome-associated responses towards prebiotic interventions and the need to fine-tune personalized prebiotic interventions and improve efficacy rates beyond a one-size-fits-all approach. Thus, greater opportunities for successful clinical outcomes can be achieved in dietary intervention studies by genotyping baseline microbiomes prior to assigning individuals to their respective treatments.

Nevertheless, we recognize that larger sample sizes will be necessary for more rigorous testing of the described framework. In addition, the prebiotics used in this study had relatively simple structures, and predictive models for structurally complex substrates may require more experimental data. Furthermore, biochemical experiments and transcriptomics will be needed to validate the mechanistic capture and metabolism of substrates by these genes and to establish that they are actively expressed in the presence of the prebiotic substrate.

## Conclusions

In this study, we combined in vitro fecal enrichments, shotgun metagenomics, and machine learning to identify substrate-specific gene signatures that could predict prebiotic responders and non-responders. The use of in vitro fecal fermentation systems provided a basis for assessing changes in the microbiome and relevant metabolic markers to distinguish responses across different prebiotic interventions. In particular, we used microbial carbohydrate metabolism genes as features to personalize prebiotic recommendations and importantly, we validated these predictions in a pilot human feeding study. The panel of predictive features could be functionalized by incorporation into a rapid PCR-based diagnostic test for real-time dietary recommendations in clinical spaces. Additionally, this microbiome approach can be scaled and integrated into screening pipelines for the discovery, design, and development of nutraceuticals beyond prebiotics, aimed at personalized responses.

## Experimental procedures

### Fecal sample collection and processing for in vitro fermentations

Fecal samples were collected from 40 adult volunteers. The following criteria were used to determine participant eligibility; (i) has no known gastrointestinal disease, (ii) is at least 19 years of age, (iii) have not consumed antibiotics or probiotic supplements in the last 3 months and (iv) is not a regular consumer of yogurt. This study was approved by the University of Nebraska-Lincoln Institutional Review Board (IRB No. 20,160,616,139). All participants provided written informed consent prior to performing study protocols.

All participants were provided with a commode specimen collection kit (Fisher Scientific, NH, USA) along with ice packs for sample collection and preservation. Samples were processed immediately after collection in an anaerobic chamber (Bactron IV anaerobic chamber; Sheldon Manufacturing, Cornelius, OR, USA) (5% H2, 5% CO2, 90% N2). Samples were weighed, diluted (1:10) and homogenized in phosphate-buffered-saline (PBS) with 10% glycerol at pH 7. Subsequently, samples were filtered with cheese cloth, and these fecal slurries were stored at -80 °C. Follow-up questionnaires were distributed to subjects after sample collection to obtain information corresponding to age, sex and broad dietary preferences. Out of 40 subjects, 36 responded to the follow up survey (Table S1).

### In vitro fecal fermentations

Stepwise in vitro fermentations were conducted anaerobically as described in Kok et al. [75] with slight modifications. Prebiotic substrate of high purities (> 95%) were used for the fecal fermentations; XOS (Prenexus Health, Az, USA), FOS (Orafti P95, Beneo, Belgium) and inulin (Orafti HP, Beneo). Each fecal sample was treated as an individual experimental unit and duplicate fermentations were performed for each fecal-prebiotic combination. Negative controls, with fermentations conducted in the absence of a prebiotic substrate, were done in parallel. Fecal slurries (3.0 ml) were thawed and then inoculated into 6.0 ml of a peptone-based fermentation media [76] containing prebiotic substrates at a final concentration of 0.5%. Every 12 h, 100-fold dilutions were performed by transferring 100 ul of fermentation sample into 9.9 ml of fresh fermentation broth. Each fermentation was continued for a total of 48 h that included 3 transfers. In addition, a spike-in of the same fecal origin was introduced after 24 h at a 1:9 ratio (v/v) in an effort to maintain competition. Samples were collected after each transfer and stored at -80 °C for subsequent downstream analyses.

### Carbohydrate quantification and metabolite analyses

Fecal fermentation samples were centrifuged at 8,000 x g for 5 min, and supernatants were collected and used for downstream analyses that included SCFA quantification, quantification of total carbohydrates, and pH. SCFA concentrations were measured using gas chromatography with a flame ionization detector as described in Yang and Rose [76]. The extraction procedure was carried out as described in Kok et al. [75]. An internal standard of 2-ethylbutyrate was used for standardization across all samples. Total carbohydrates were quantified with a phenol-sulfuric acid microplate assay as described in Medina et al. [77]. Standard curves were made from a dilution series of xylose and fructose, starting at a concentration of 0.1 mg/ml. The absorbance was measured using a plate reader (BioTek Synergy H1, Winooski, Vermont, USA) at 490 nm. Supernatant pH was measured using a standard pH meter (Orion Research Inc., Boston, Mass., USA) with a micro combination electrode (Mettler Toledo LE422, Columbus, Ohio).

### DNA extraction, metagenomic sequencing and analyses

DNA was extracted using phenol-chloroform as described in Martinez et al. [78]. DNA was resuspended in DNAse-free water. Illumina metagenomic libraries were prepared using an NEBNext Ultra II FS DNA Library Prep Kit (New England Biolabs, Ipswich, MA, USA) and frozen DNA samples were sent to Novogene Co. Ltd (Sacramento, CA, USA) for shotgun metagenomic sequencing on an Illumina HiSeq platform (2 × 150 bp). Reads were removed if they contained adapters, contained > 10% of undetermined bases, had a phred score of lower than 25, or were of host contaminant, which resulted in an average of 12.3 million reads per sample. Rarefactions curves were plotted to assess the impact of sequencing depth on the number of predicted genes. Reads were subsampled at different depths (0.1, 0.5, 1, 2, 3, 4, 5, 6, 7, 8, 9, 10 million reads) and the number of predicted coding genes were determined for each subsample using Prokka [79].

Taxonomic profiling of metagenomic reads were carried out with MetaPhlAn2 [80]. To avoid fragmented assemblies, reads were firstly assembled individually using Megahit [81] and then merged. Duplicates and contigs shorter than 2000 bp were removed. The resulting longer contigs were co-assembled with individual reads using metaSPAdes [82] and the final assembly was evaluated using metaQuast [83]. Reads were mapped back to the assembly to determine read coverage using bamtools [84]. The Prokka rapid annotation tool [79] and eggNOG-mapper was used to annotate genes in the assembly. dbCAN2 [85]was used to identify CAZymes as well as CGCs. These clusters included CAZymes that were present together with at least one transporter and with an intergenic distance threshold of 5 non-signature genes. Additional annotation was performed using a DIAMOND search [86] against the NCBI non-redundant sequence database. Salmon was used to quantify abundances of gene clusters across all samples [87], and DESeq2 [88] was used to identify DA CGCs between responders and non-responders for each prebiotic. Phobius [89] was used to predict transmembrane topology and the presence of signal peptides in genes.

### Statistical analyses and machine learning

All statistical analyses were carried out in R (version 4.1.1). Using the bestNormalize package [90], an ordered quantile normalized transformation was used to transform SCFA concentrations to satisfy modelling assumptions for normality. No significant outliers were detected using the identify outliers helper function in the rstatix package [91]that adopts boxplot methods to identify univariate outliers. Then, a two-way repeated measure ANOVA was used to evaluate significant differences in total acetate and butyrate concentrations using prebiotic treatment and time as factors while accounting for within subject dependence. As there were no significant interactions between prebiotic and time, the means of all time points of duplicate experiments were jointly considered when phenotyping subjects as responders and non-responders.

One-way ANOVA was used to compare differences in total acetate and butyrate concentrations between treatments within each subject, followed by pairwise t-test. Post-hoc Dunnett’s test was then used to compare each prebiotic treatment with the no-prebiotic control to determine responder and non-responder phenotypes. Overall significant differences in the sum of acetate and butyrate concentrations between responders and non-responders per prebiotic substrate were determined using a t-test. Wilcoxon tests were used to determine significant differences in taxonomic relative abundances and gene copy number between responders and non-responders. Chi-square goodness of fit tests were used to determine differences in the number of differential abundant genes between responders and non-responders. Paired t-tests were used to compare SCFA concentrations, *Bifidobacterium* counts and prebiotic gene cluster copy numbers before and after prebiotic consumption in the human feeding trial. One-way ANOVA tests were used to compare each dietary component/category between treatments as reported in the dietary history questionnaire (DHQ3). Friedman test was used to compare changes in gastrointestinal symptoms between visits within subjects. Spearman correlations were used to determine significant correlations between SCFA production with pH, carbohydrate consumption, and taxonomic abundances derived from metagenomic sequences. Holm-Bonferroni adjustments were used for multiple comparisons and FDR adjustments were used for multiple hypotheses testing.

The mikropml package [92] was used to create RBF SVM classification models for each of the three prebiotics and for feature permutation and feature selection. Due to sample size limitation, a random partition seed was used to split the dataset into training and testing sets at a ratio of 50:50. The training set was used to tune and train the model while the testing set was withheld for unbiased evaluation of model performance. Specifically, a grid search was used to tune hyperparameters and estimate the optimal cost (C) and sigma (σ) parameters and 100 times repeated 5-fold cross validation was used to train the model. Model performances on both training and test sets were evaluated using measures such as sensitivity, specificity, and the area under the receiver operating characteristic curve (AUC-ROC). Feature permutation was carried out to identify discriminatory features between responders and non-responders. Features are ranked based on mean differences between test and permuted AUC. A larger difference indicates a higher feature contribution towards model robustness. Phenotype predictions were assigned using the stats package in R with quantified gene copy numbers as features.

### Quantification of *Bifidobacterium* and prebiotic candidate genes using quantitative PCR (qPCR)

Quantification of *Bifidobacterium* in baseline and post-fermentation (48 h) samples were performed by qPCR as described previously [93] using genus-specific primers (5’-TCGCGTCYGGTGTGAAAG-3’, forward and 5’-CCACATCCAGCRTCCAC-3’, reverse). A standard curve was made using 10-fold serial dilutions of genomic DNA isolated from a pure culture of *Bifidobacterium longum*. Cycle threshold (C_T_) values were plotted against log_10_ CFU/ml values for quantification. For targeted prebiotic-specific genes in the previously identified CGCs, qPCR primers were designed using RUCS [94]. *In silico* validation was performed to ensure primer specificity (emboss primersearch), followed by Sanger sequencing of PCR products. qPCR reactions were performed using a QuantStudio 5 Real-Time PCR system (ThermoFisher Scientific, Waltham, MA, USA). Each reaction mixture contained 5ul of qPCR Master Mix (2X Maxima SYBR green; ThermoFisher Scientific, Waltham, MA, USA), 1 μm of a forward and reverse primer, 3.5 ul of water and 0.5ul of DNA template for a total volume of 10 ul reactions per well. An annealing temperature of 60 °C was used for all primers. Standard curves were made using 10-fold dilutions of purified PCR products and concentrations of PCR products were measured using a Qubit fluorometer. The number of gene copies of the purified PCR products were calculated using the following formula:

$$\begin{array}{l}gene\,copies\, = \,\left( {DNA\,concentration\,\left[ {\frac{{ng}}{{ul}}} \right]} \right)\left( {\frac{{1g}}{{1,{{000}^3}ng}}} \right)\left( {\frac{{1\,mol\,bp\,DNA}}{{660g\,DNA}}} \right)\\\times \left( {\frac{{6.023 \times {{10}^2}^3bp}}{{molbp}}} \right)\left( {\frac{{1\,copy}}{{PCR\,product\,size\,[bp]}}} \right) \times volume\,of\,template\,(ul)\end{array}$$

Then, log_10_ copy number values were plotted against C_T_ values to obtain a standard curve. Baseline pre-fermentation samples and cDNA samples were then screened for the presence of these genes and quantified according to the standard curves.

### Human feeding trial

A human prebiotic feeding trial was conducted using a single-blind, multi-arm parallel-group stratified design. Subjects were enrolled with the following eligibility criteria: (i) no known gastrointestinal disease; (ii) is at least 19 years of age; (iii) have not consumed antibiotics in the last 6 months; and (iv) is not a regular consumer of probiotic supplements. This study was approved by the University of Nebraska-Lincoln Institutional Review Board (IRB #20210821276FB). All subjects provided written informed consent prior to enrolment.

Enrolment was conducted on a rolling basis and each participant delivered three fecal samples to the clinical facility. The first run-in fecal sample (visit 1) was screened for genes of interest and to predict response status to each of the test prebiotics, FOS, XOS, and inulin. The previously trained SVM models were used for these predictions, and prebiotic gene profiles were generated for each subject. In instances where subjects were predicted responders to two or more prebiotics, subjects were randomly stratified such that treatment groups were balanced with a near equal number of samples. Subjects who were predicted non-responders to all prebiotics were given XOS (5 g per day) and were used as a null response control group in the study. Participants were stratified as follows; 7 XOS responders, 7 FOS responders, 5 inulin responders and 8 non-responders. Participants were also asked to complete a dietary history questionnaire online at visit 1. Participants begin the feeding study (5 g of each prebiotic) after a period of two-three weeks from the first visit. At visit 2, participants provided another fecal sample and were asked to complete a gastrointestinal symptom questionnaire. At this visit, participants were also given the test prebiotics and were instructed to consume the prebiotics for a period of two weeks. After two weeks, participants provided a third fecal sample and were asked to complete another gastrointestinal symptom questionnaire. Fecal sample collection and processing (DNA extraction and SCFA measurements) of all samples across all visits (*n* = 81) is as described above for in vitro fermentations. Frozen DNA samples were sent to Novogene Co. Ltd (Sacramento, CA, USA) for V3-V4 16 S rRNA sequencing with an Illumina NovaSeq 6000 (2 × 250 bp). 16 S rRNA sequences were analysed using QIIME2 and sequences were dereplicated into unique amplicon sequence variants (ASV) with DADA2 [95, 96]. Taxonomy was assigned to representative sequences using a pretrained classifier of the SILVA database (v138) based on 99% sequence identity. Downstream statistical analysis was carried out in RStudio (ver 4.2.2) with the phyloseq package [97].

### Electronic supplementary material

Below is the link to the electronic supplementary material.

**Additional File 1**. Supplementary Figures S1-S5

**Additional File 2**. Supplementary Tables S1-S12

## Data Availability

Metagenomic sequences are available in NCBI (accession no. PRJNA823736).

## Abbreviations

FOS: fructooligosaccharide
XOS: xylooligosaccharide
SCFA: short chain fatty acids
CAZymes: carbohydrate active enzymes
GH: glycosyl hydrolase
CGC: carbohydrate gene clusters
RBF: Radial Basis Function
ASBC: ATP-binding cassette
XCG: XOS gene cluster
FCG: FOS gene cluster
ICG: Inulin gene cluster
SVM: Support Vector Machine
AUC: area under the curve

## References

[1] Johnson AJ, Vangay P, Al-Ghalith GA, Hillmann BM, Ward TL, Shields-Cutler RR (2019). Daily sampling reveals personalized diet-microbiome associations in humans. Cell Host Microbe.

[2] Maldonado-Gómez MX, Martínez I, Bottacini F, O’Callaghan A, Ventura M, van Sinderen D (2016). Stable engraftment of Bifidobacterium longum AH1206 in the human gut depends on individualized features of the resident microbiome. Cell Host Microbe.

[3] David LA, Materna AC, Friedman J, Campos-Baptista MI, Blackburn MC, Perrotta A et al. Host lifestyle affects human microbiota on daily timescales. Genome Biol. 2015;15. 10.1186/gb-2014-15-7-r89 PMC4405912 25146375

[4] Kovatcheva-Datchary P, Nilsson A, Akrami R, Lee YS, de Vadder F, Arora T (2015). Dietary fiber-induced improvement in glucose metabolism is associated with increased abundance of Prevotella. Cell Metab.

[5] Rodriguez J, Hiel S, Neyrinck AM, Le Roy T, Pötgens SA, Leyrolle Q (2020). Discovery of the gut microbial signature driving the efficacy of prebiotic intervention in obese patients. Gut.

[6] Nguyen NK, Deehan EC, Zhang Z, Jin M, Baskota N, Perez-Muñoz ME et al. Gut microbiota modulation with long-chain corn bran arabinoxylan in adults with overweight and obesity is linked to an individualized temporal increase in fecal propionate. Microbiome. 2020;8. 10.1186/s40168-020-00887-w PMC7439537 32814582

[7] Zeevi D, Korem T, Zmora N, Halpern Z, Elinav E, Correspondence ES (2015). Personalized nutrition by prediction of glycemic responses. Cell.

[8] Berry SE, Valdes AM, Drew DA, Asnicar F, Mazidi M, Wolf J (2020). Human postprandial responses to food and potential for precision nutrition. Nat Med.

[9] Gibson R, Hutkins R, Sanders M, Al E (2017). Expert consensus document: the International Scientific Association for Probiotics and Prebiotics (ISAPP) consensus statement on the definition and scope of prebiotics. Nat Rev Gastroenterol Hepatol.

[10] Clayton TA, Baker D, Lindon JC, Everett JR, Nicholson JK (2009). Pharmacometabonomic identification of a significant host-microbiome metabolic interaction affecting human drug metabolism. Proc Natl Acad Sci USA.

[11] Volokh O, Klimenko N, Berezhnaya Y, Tyakht A, Nesterova P, Popenko A et al. Human gut microbiome response induced by fermented dairy product intake in healthy volunteers. Nutrients. 2019;11. 10.3390/nu11030547 PMC6470569 30836671

[12] Martínez I, Kim J, Duffy PR, Schlegel VL, Walter J (2010). Resistant starches types 2 and 4 have differential effects on the composition of the fecal microbiota in human subjects. PLoS ONE.

[13] Krumbeck JA, Rasmussen HE, Hutkins RW, Clarke J, Shawron K, Keshavarzian A et al. Probiotic Bifidobacterium strains and galactooligosaccharides improve intestinal barrier function in obese adults but show no synergism when used together as synbiotics. Microbiome. 2018;6. 10.1186/s40168-018-0494-4 PMC6022452 29954454

[14] Davis L, Martínez I, Walter J, Goin C, Hutkins RW (2011). Barcoded pyrosequencing reveals that consumption of galactooligosaccharides results in a highly specific bifidogenic response in humans. PLoS ONE.

[15] Ramos-Romero S, Léniz A, Martínez-Maqueda D, Amézqueta S, Fernández-Quintela A, Hereu M et al. Inter-individual variability in insulin response after grape pomace supplementation in subjects at high cardiometabolic risk: role of microbiota and miRNA. Mol Nutr Food Res. 2021;65. 10.1002/mnfr.202000113 33202108

[16] Cantu-Jungles TM, Hamaker BR (2020). New view on dietary fiber selection for predictable shifts in gut microbiota. mBio.

[17] Ryu G, Kim H, Koh A (2021). Approaching precision medicine by tailoring the microbiota. Mamm Genome.

[18] Loomba R, Seguritan V, Li W, Long T, Klitgord N, Bhatt A (2017). Gut microbiome-based metagenomic signature for non-invasive detection of advanced fibrosis in human nonalcoholic fatty liver disease. Cell Metab.

[19] Gupta VK, Kim M, Bakshi U, Cunningham KY, Davis JM, Lazaridis KN et al. A predictive index for health status using species-level gut microbiome profiling. Nat Commun. 2020;11. 10.1038/s41467-020-18476-8 PMC7492273 32934239

[20] Leeming ER, Louca P, Gibson R, Menni C, Spector TD, Le Roy CI. The complexities of the diet-microbiome relationship: advances and perspectives. Genome Med. 2021;13. 10.1186/s13073-020-00813-7 PMC7819159 33472701

[21] Korpela K, Flint HJ, Johnstone AM, Lappi J, Poutanen K, Dewulf E (2014). Gut microbiota signatures predict host and microbiota responses to dietary interventions in obese individuals. PLoS ONE.

[22] Selak M, Rivière A, Moens F, Van den Abbeele P, Geirnaert A, Rogelj I (2016). Inulin-type fructan fermentation by bifidobacteria depends on the strain rather than the species and region in the human intestine. Appl Microbiol Biotechnol.

[23] Zhu A, Sunagawa S, Mende DR, Bork P (2015). Inter-individual differences in the gene content of human gut bacterial species. Genome Biol.

[24] Johnson JS, Spakowicz DJ, Hong BY, Petersen LM, Demkowicz P, Chen L et al. Evaluation of 16S rRNA gene sequencing for species and strain-level microbiome analysis. Nat Commun. 2019;10. 10.1038/s41467-019-13036-1 PMC6834636 31695033

[25] Deehan EC, Yang C, Perez-Muñoz ME, Nguyen NK, Cheng CC, Triador L et al. Precision microbiome modulation with discrete dietary fiber structures directs short-chain fatty acid production. Cell Host Microbe. 2020;27. 10.1016/j.chom.2020.01.006 32004499

[26] Filippis F, De, Pasolli E, Tett A, Tarallo S, Naccarati A, Angelis M, De (2019). Distinct genetic and functional traits of human intestinal Prevotella copri strains are associated with different habitual diets. Cell Host Microbe.

[27] Flint HJ, Scott KP, Duncan SH, Louis P, Forano E (2012). Microbial degradation of complex carbohydrates in the gut. Gut Microbes.

[28] Ndeh D, Gilbert HJ (2018). Biochemistry of complex glycan depolymerisation by the human gut microbiota. FEMS Microbiol Rev.

[29] Sheridan PO, Martin JC, Lawley TD, Browne HP, Harris HMB, Bernalier-Donadille A (2015). Polysaccharide utilization loci and nutritional specialization in a dominant group of butyrate-producing human colonic Firmicutes. Microb Genom.

[30] Wexler AG, Goodman AL. An insider’s perspective: Bacteroides as a window into the microbiome. Nat Microbiol. 2017;2. 10.1038/nmicrobiol.2017.26 PMC5679392 28440278

[31] Kundi ZM, Lee JC-Y, Pihlajamäki J, Chan CB, Leung KS, So SSY et al. Dietary fiber from oat and rye brans ameliorate western diet–induced body weight gain and hepatic inflammation by the modulation of short-chain fatty acids, bile acids, and tryptophan metabolism. Mol Nutr Food Res. 2021;65. 10.1002/mnfr.201900580 32526796

[32] Tanes C, Bittinger K, Gao Y, Friedman ES, Nessel L, Paladhi UR (2021). Role of dietary fiber in the recovery of the human gut microbiome and its metabolome. Cell Host Microbe.

[33] Yoshida K, Hirano R, Sakai Y, Choi M, Sakanaka M, Kurihara S et al. Bifidobacterium response to lactulose ingestion in the gut relies on a solute-binding protein-dependent ABC transporter. Commun Biol. 2021;4. 10.1038/s42003-021-02072-7 PMC8110962 33972677

[34] Holmes ZC, Silverman JD, Dressman HK, Wei Z, Dallow EP, Armstrong SC (2020). Short-chain fatty acid production by gut microbiota from children with obesity differs according to prebiotic choice and bacterial community composition. mBio.

[35] Algera JP, Magnusson MK, Öhman L, Störsrud S, Simrén M, Törnblom H (2022). Randomised controlled trial: effects of gluten-free diet on symptoms and the gut microenvironment in irritable bowel syndrome. Aliment Pharmacol Ther.

[36] Cheng R, Wang L, Le S, Yang Y, Zhao C, Zhang X (2022). A randomized controlled trial for response of microbiome network to exercise and diet intervention in patients with nonalcoholic fatty liver disease. Nat Commun 2022.

[37] Rej A, Sanders DS, Shaw CC, Buckle R, Trott N, Agrawal A (2022). Efficacy and acceptability of Dietary therapies in Non-constipated irritable bowel syndrome: a Randomized Trial of Traditional Dietary advice, the low FODMAP Diet, and the Gluten-Free Diet. Clinl Gastroenterol Hepatol.

[38] Reid G, Gaudier E, Guarner F, Huffnagle GB, Macklaim JM, Munoz AM et al. Responders and non-responders to probiotic interventions: how can we improve the odds? Gut Microbes. 2010;1. 10.4161/gmic.1.3.12013 PMC3023600 21637034

[39] Ojima MN, Yoshida K, Sakanaka M, Jiang L, Odamaki T, Katayama T (2022). Ecological and molecular perspectives on responders and non-responders to probiotics and prebiotics. Curr Opin Biotechnol.

[40] Blaak EE, Canfora EE, Theis S, Frost G, Groen AK, Mithieux G (2020). Short chain fatty acids in human gut and metabolic health. Benef Microbes.

[41] Davis L, Martínez I, Walter J, Hutkins R (2010). A dose dependent impact of prebiotic galactooligosaccharides on the intestinal microbiota of healthy adults. Int J Food Microbiol.

[42] Topping DL, Clifton PM (2001). Short-chain fatty acids and human colonic function: roles of resistant starch and nonstarch polysaccharides. Physiol Rev.

[43] Oliver A, Chase AB, Weihe C, Orchanian SB, Riedel SF, Hendrickson CL (2021). High-fiber, whole-food dietary intervention alters the human gut microbiome but notgecal short-chain fatty acids. mSystems.

[44] Rahman MN, Diantini A, Fattah M, Barliana MI, Wijaya A (2021). A highly sensitive, simple, and fast gas chromatography–mass spectrometry method for the quantification of serum short-chain fatty acids and their potential features in central obesity. Anal Bioanal Chem.

[45] Neyrinck AM, Rodriguez J, Zhang Z, Nazare JA, Bindels LB, Cani PD (2022). Breath volatile metabolome reveals the impact of dietary fibres on the gut microbiota: Proof of concept in healthy volunteers. EBioMedicine.

[46] Lee JHJ, Zhu J (2021). Analyses of short-chain fatty acids and exhaled breath volatiles in dietary intervention trials for metabolic diseases. Exp Biol Med.

[47] Dalile B, Vervliet B, Bergonzelli G, Verbeke K, van Oudenhove L (2020). Colon-delivered short-chain fatty acids attenuate the cortisol response to psychosocial stress in healthy men: a randomized, placebo-controlled trial. Neuropsychopharmacol 2020.

[48] Gurry T, Nguyen LTT, Yu X, Alm EJ. Functional heterogeneity in the fermentation capabilities of the healthy human gut microbiota. PLoS ONE. 2021;16. 10.1371/journal.pone.0254004 PMC8294568 34288919

[49] Flint HJ, Bayer EA, Rincon MT, Lamed R, White BA (2008). Polysaccharide utilization by gut bacteria: potential for new insights from genomic analysis. Nat Rev Microbiol.

[50] Cecchini DA, Laville E, Laguerre S, Robe P, Leclerc M, Doré J (2013). Functional metagenomics reveals novel pathways of prebiotic breakdown by human gut bacteria. PLoS ONE.

[51] Liu S, Fang Z, Wang H, Zhai Q, Hang F, Zhao J et al. Gene – phenotype associations involving human-residential bifidobacteria (HRB) reveal significant species- and strain-specificity in carbohydrate catabolism. Microorganisms. 2021;9. 10.3390/microorganisms9050883 PMC8143103 33919102

[52] Briggs JA, Grondin JM, Brumer H (2021). Communal living: glycan utilization by the human gut microbiota. Environ Microbiol.

[53] Hamaker BR, Tuncil YE (2014). A perspective on the complexity of dietary fiber structures and their potential effect on the gut microbiota. J Mol Biol.

[54] Kelly SM, Munoz-Munoz J, van Sinderen D. Plant glycan metabolism by Bifidobacteria. Front Microbiol. 2021;12. 10.3389/fmicb.2021.609418 PMC7889515 33613480

[55] Bolam DN, Sonnenburg JL. Mechanistic insight into polysaccharide use within the intestinal microbiota. Gut Microbes. 2011;2. 10.4161/gmic.2.2.15232 PMC3225772 21637023

[56] Joglekar P, Sonnenburg ED, Higginbottom SK, Earle KA, Morland C, Shapiro-Ward S et al. Genetic varation of the SusC/SusD homologs from a polysaccharide utilization locus underlies divergent fructan specificities and functional adaptation in Bacteroides thetaiotaomicron strains. mSphere. 2018;3. 10.1128/mSphereDirect.00185-18 PMC5967196 29794055

[57] Rakoff-Nahoum S, Foster KR, Comstock LE (2016). The evolution of cooperation within the gut microbiota. Nature.

[58] Leth ML, Ejby M, Workman C, Ewald DA, Pedersen SS, Sternberg C (2018). Differential bacterial capture and transport preferences facilitate co-growth on dietary xylan in the human gut. Nat Microbiol 2018.

[59] Saito Y, Shigehisa A, Watanabe Y, Tsukuda N, Moriyama-Ohara K, Hara T et al. Multiple transporters and glycoside hydrolases are involved in arabinoxylan derived oligosaccharide utilization in Bifidobacterium pseudocatenulatum. Appl Environ Microbiol. 2020;86. 10.1128/AEM.01782-20 PMC7688211 33036985

[60] Zhang M, Chekan JR, Dodd D, Hong P-Y, Radlinski L, Revindran V (2014). Xylan utilization in human gut commensal bacteria is orchestrated by unique modular organization of polysaccharide-degrading enzymes. Proc Natl Acad Sci USA.

[61] Cohen Y, Borenstein E (2022). The microbiome’s fiber degradation profile and its relationship with the host diet. BMC Biol.

[62] Tian L, Wang X-W, Wu A-K, Fan Y, Friedman J, Dahlin A (2020). Deciphering functional redundancy in the human microbiome. Nat Commun 2020.

[63] Moya A, Ferrer M (2016). Functional redundancy-Induced Stability of Gut Microbiota subjected to Disturbance. Trends Microbiol.

[64] Ma C, Wasti S, Huang S, Zhang Z, Mishra R, Jiang S (2020). The gut microbiome stability is altered by probiotic ingestion and improved by the continuous supplementation of galactooligosaccharide. Gut Microbes.

[65] Ravi A, Troncoso-Rey P, Ahn-Jarvis J, Corbin KR, Harris S, Harris H et al. Linking carbohydrate structure with function in the human gut microbiome using hybrid metagenome assemblies. bioRxiv. 2021. 10.1038/s42003-022-03865-0 PMC9458734 36076058

[66] Rampelli S, Schnorr SL, Consolandi C, Turroni S, Severgnini M, Peano C (2015). Metagenome sequencing of the Hadza hunter-gatherer gut microbiota. Curr Biol.

[67] Soverini M, Turroni S, Biagi E, Quercia S, Brigidi P, Candela M et al. Variation of carbohydrate-active enzyme patterns in the gut microbiota of Italian healthy subjects and type 2 diabetes patients. Front Microbiol. 2017;8. 10.3389/fmicb.2017.02079 PMC5660705 29114246

[68] Accetto T, Avguštin G (2015). Polysaccharide utilization locus and CAZYme genome repertoires reveal diverse ecological adaptation of Prevotella species. Syst Appl Microbiol.

[69] Rodriguez CI, Martiny JBH. Evolutionary relationships among bifidobacteria and their hosts and environments. BMC Genomics. 2020;21. 10.1186/s12864-019-6435-1 PMC6950798 31914919

[70] Sonnenburg JL, Xu J, Leip DD, Chen C-H, Westover BP, Weatherford J et al. Glycan foraging in vivo by an intestine-adapted bacterial symbiont. Science (1979). 2005;307. 10.1126/science.1109051 15790854

[71] Koropatkin NM, Cameron EA, Martens EC (2012). How glycan metabolism shapes the human gut microbiota. Nat Rev Microbiol.

[72] Broekaert WF, Courtin CM, Verbeke K, Wiele T, Van de, Verstraete W, Delcour JA (2011). Prebiotic and other health-related effects of cereal-derived arabinoxylans, arabinoxylan-oligosaccharides, and xylooligosaccharides. Crit Rev Food Sci Nutr.

[73] Garrido D, Kim JH, German JB, Raybould HE, Mills DA (2011). Oligosaccharide binding proteins from Bifidobacterium longum subsp. infantis reveal a preference for host glycans. PLoS ONE.

[74] Mueller M, Reiner J, Fleischhacker L, Viernstein H, Loeppert R, Praznik W (2016). Growth of selected probiotic strains with fructans from different sources relating to degree of polymerization and structure. J Funct Foods.

[75] Kok CR, Quintero DFG, Niyirora C, Rose D, Li A, Hutkins R (2019). An in vitro enrichment strategy for formulating synergistic synbiotics. Appl Environ Microbiol.

[76] Yang J, Rose DJ (2014). Long-term dietary pattern of fecal donor correlates with butyrate production and markers of protein fermentation during in vitro fecal fermentation. Nutr Res.

[77] Medina DA, Pinto F, Ovalle A, Thomson P, Garrido D. Prebiotics mediate microbial interactions in a consortium of the infant gut microbiome. Int J Mol Sci. 2017;18. 10.3390/ijms18102095 PMC5666777 28976925

[78] Martínez I, Stegen JC, Maldonado-Gómez MX, Eren AM, Siba PM, Greenhill AR (2015). The gut microbiota of rural Papua New guineans: composition, diversity patterns, and ecological processes. Cell Rep.

[79] Seemann T (2014). Prokka: rapid prokaryotic genome annotation. Bioinformatics.

[80] Truong DT, Franzosa EA, Tickle TL, Scholz M, Weingart G, Pasolli E (2015). MetaPhlAn2 for enhanced metagenomic taxonomic profiling. Nat Methods 2015.

[81] Li D, Liu CM, Luo R, Sadakane K, Lam TW (2015). MEGAHIT: an ultra-fast single-node solution for large and complex metagenomics assembly via succinct de bruijn graph. Bioinformatics.

[82] Nurk S, Meleshko D, Korobeynikov A, Pevzner PA (2017). MetaSPAdes: a new versatile metagenomic assembler. Genome Res.

[83] Mikheenko A, Saveliev V, Gurevich A, MetaQUAST (2016). Evaluation of metagenome assemblies. Bioinformatics.

[84] Barnett DW, Garrison EK, Quinlan AR, Str̈mberg MP, Marth GT (2011). Bamtools: a C + + API and toolkit for analyzing and managing BAM files. Bioinformatics.

[85] Zhang H, Yohe T, Huang L, Entwistle S, Wu P, Yang Z (2018). dbCAN2: a meta server for automated carbohydrate-active enzyme annotation. Nucleic Acids Res.

[86] Buchfink B, Xie C, Huson DH (2014). Fast and sensitive protein alignment using DIAMOND. Nat Methods.

[87] Patro R, Duggal G, Love MI, Irizarry RA, Kingsford L. Salmon provides fast and bias-aware quantification of transcript expression. Nat Methods. 2017;14:417–19. 10.1038/nmeth.4197 PMC5600148 28263959

[88] Love MI, Huber W, Anders S (2014). Moderated estimation of Fold change and dispersion for RNA-seq data with DESeq2. Genome Biol.

[89] Käll L, Krogh A, Sonnhammer ELL (2007). Advantages of combined transmembrane topology and signal peptide prediction—the Phobius web server. Nucleic Acids Res.

[90] Peterson RA, Cavanaugh JE (2019). Ordered quantile normalization: a semiparametric transformation built for the cross-validation era. J Appl Stat.

[91] Kassambara A, rstatix. Pipe-Friendly Framework for Basic Statistical Tests. R package rstatix version 0.7.1. 2022.

[92] Topçuoğlu B, Lapp Z, Sovacool K, Snitkin E, Wiens J, Schloss P (2021). Mikropml: user-friendly R Package for supervised machine learning pipelines. J Open Source Softw.

[93] Martínez I, Wallace G, Zhang C, Legge R, Benson AK, Carr TP (2009). Diet-induced metabolic improvements in a hamster model of hypercholesterolemia are strongly linked to alterations of the gut microbiota. Appl Environ Microbiol.

[94] Christen M, Thomsen F, Hasman H, Westh H, Lya Kaya H, Lund O (2017). RUCS: rapid identification of PCR primers for unique core sequences. Bioinformatics.

[95] Bolyen E, Rideout JR, Dillon MR, Bokulich NA, Abnet CC, Al-Ghalith GA (2019). Reproducible, interactive, scalable and extensible microbiome data science using QIIME2. Nat Biotechnol.

[96] Callahan BJ, McMurdie PJ, Rosen MJ, Han AW, Johnson AJA, Holmes SP (2016). DADA2: high-resolution sample inference from Illumina amplicon data. Nat Methods.

[97] McMurdie PJ, Holmes SP (2013). Phyloseq: an R Package for Reproducible Interactive Analysis and Graphics of Microbiome Census Data. PLoS ONE.

