# Supplementary material for: Identification of carbohydrate gene clusters obtained from in vitro fermentations as predictive biomarkers of prebiotic responses
Source: BMC Microbiol. 2024 May 25;24:183. doi: 10.1186/s12866-024-03344-y (PMC11127362; doi:10.1186/s12866-024-03344-y)
Supplement: Supplementary file 1 — Additional File 1. Supplementary Figures S1-S5 [file 12866_2024_3344_MOESM1_ESM.docx]

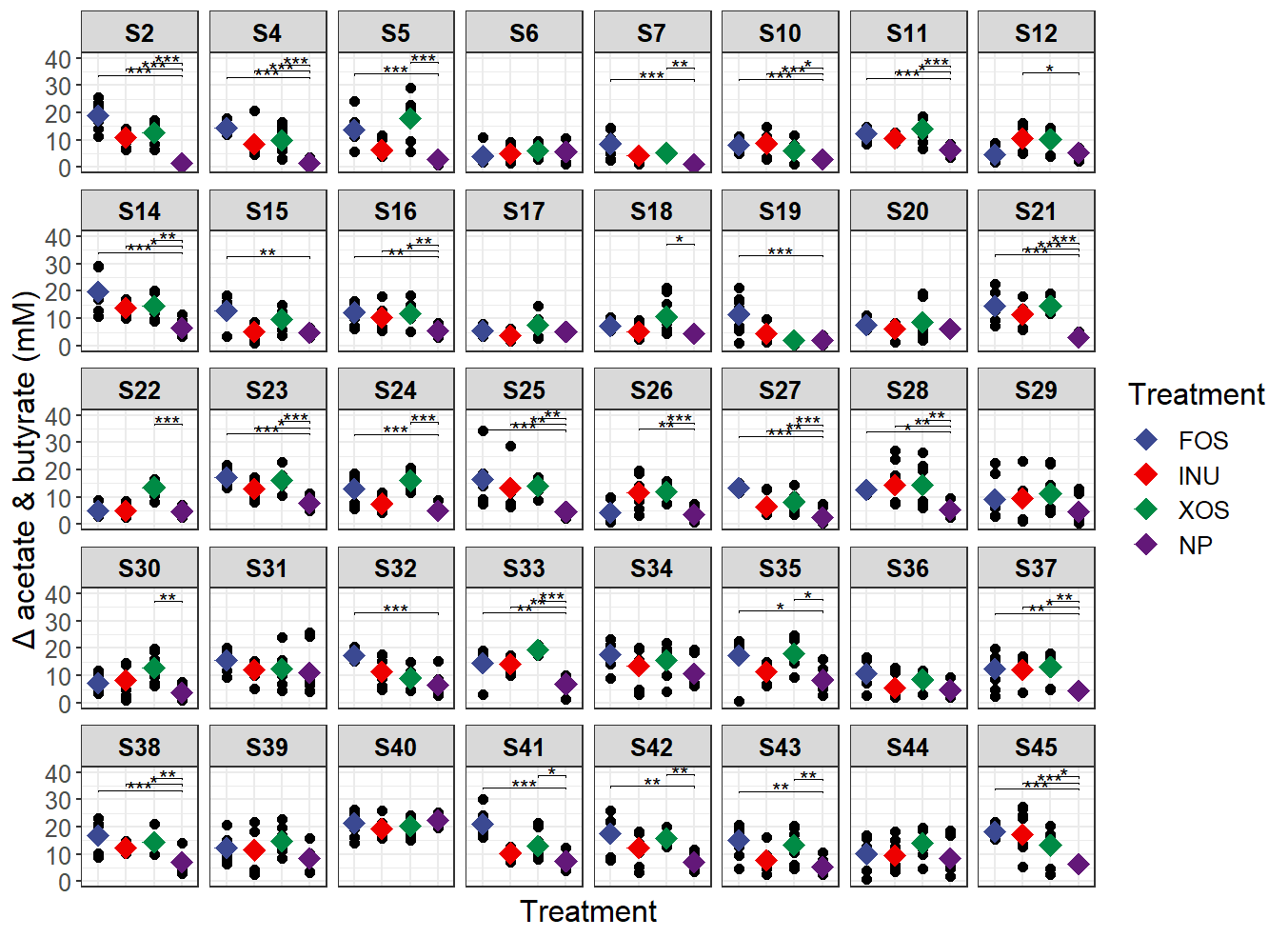


**Fig. S1: Individual SCFA profiles across prebiotic fermentations based on the sum of acetate and butyrate concentrations.** For every subject, each datapoint represents SCFA measurements at the end of every 12-hour fermentation cycle per substrate in duplicates. Baseline concentrations were subtracted from the sum of acetate and butyrate concentrations after fermentation. Dunnett’s test was used to compare SCFA concentrations of each prebiotic with parallel negative controls (NP) to determine responder and non-responder phenotypes. Black circles represent individual observations and diamonds represent the mean. Colors corresponds to each treatment; FOS: blue, inulin: red, XOS: green and NP (no prebiotic control): purple.

**B**

**A**


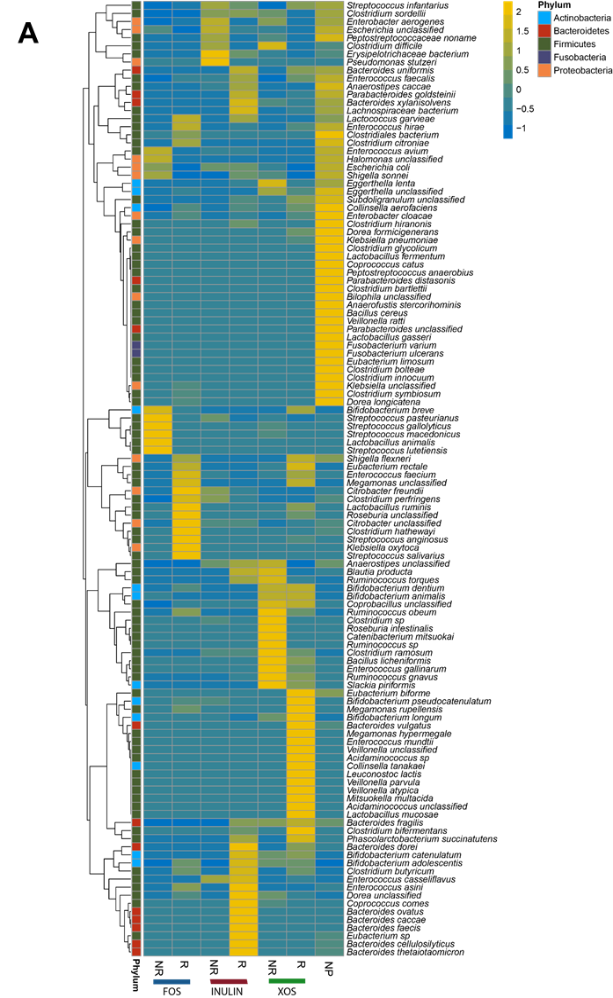


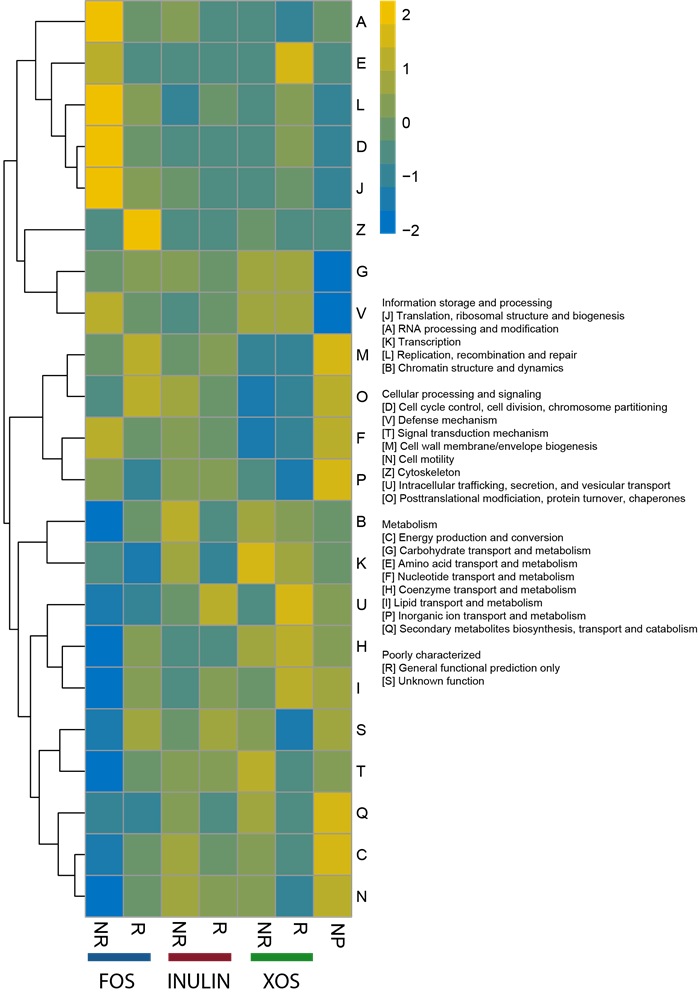


**Fig. S2: Hierarchical clustering of taxonomic species and COG functional genes from metagenomes.** A,B) Taxonomic species (A) and COG functional genes (B) were grouped based on responder and non-responder phenotypes across prebiotic substrates. Each alphabet refers to a COG function assigned according to the COG database. R: responder, NR: non-responder.


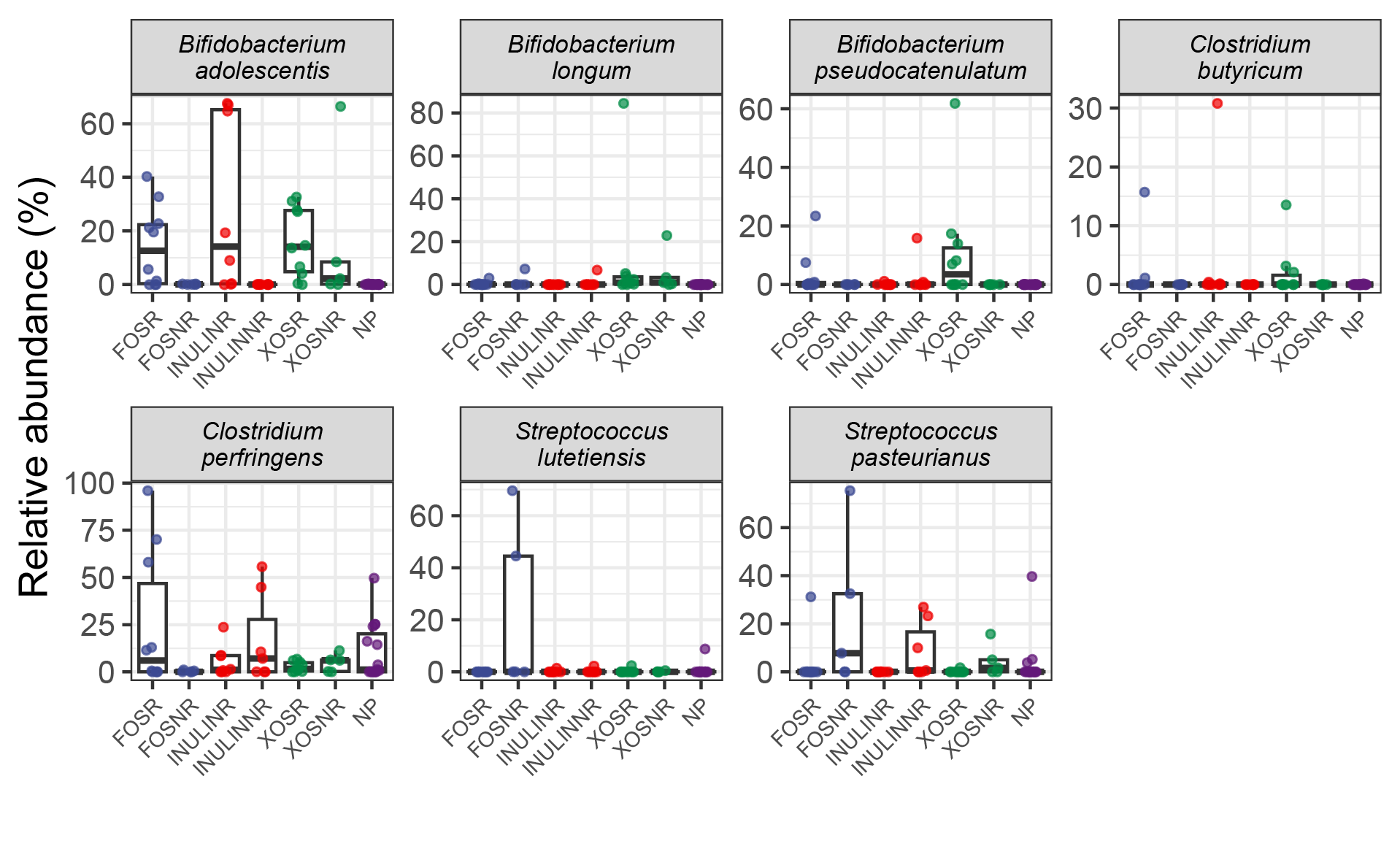


**Fig. S3: Relative abundances of selected species between responders (R) and non-responders (NR) for each prebiotic.** Each data point represents a metagenome sample. Each color represents a prebiotic treatment; FOS: blue, inulin: red, XOS: green, no-prebiotic control: NP.

**A**


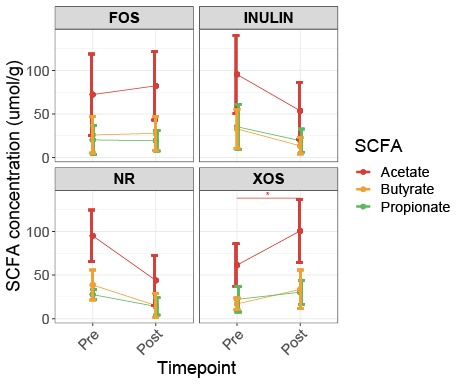


**B**


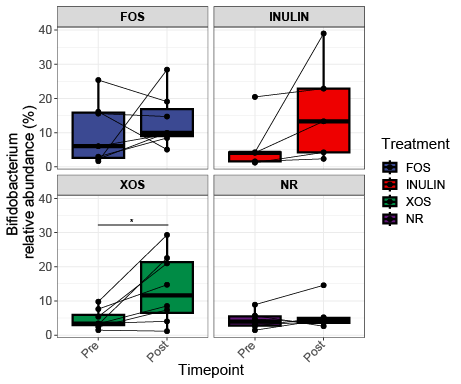


**Fig. S4: Changes in SCFA and *Bifidobacterium* relative abundance in human feeding trial.** A) Changes in total SCFA pre- and post- prebiotic feeding period as analyzed using paired t-tests (*, p<0.05). B) Changes in *Bifidobacterium* relative abundances from 16S rRNA sequencing before after the prebiotic feeding period. Statistical significance determined using paired Wilcoxon test; (*, p<0.05).


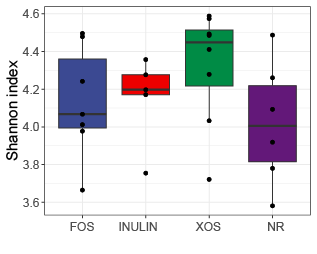
**
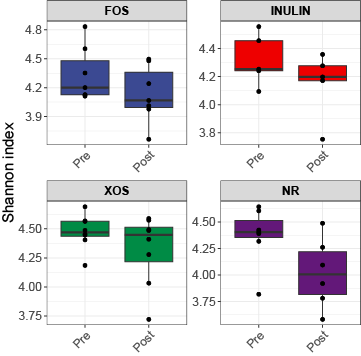
**

**A**

**B**

**C**

**D**

**E**

**F**


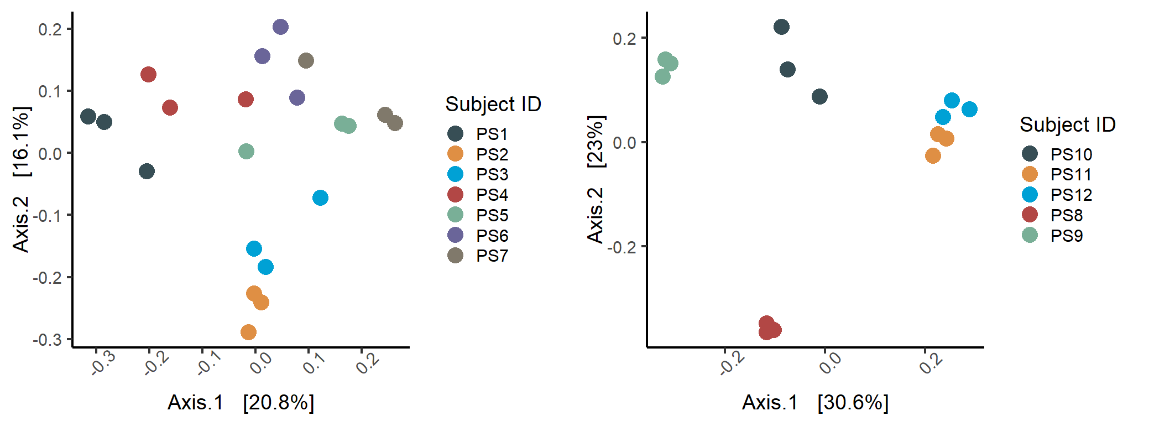


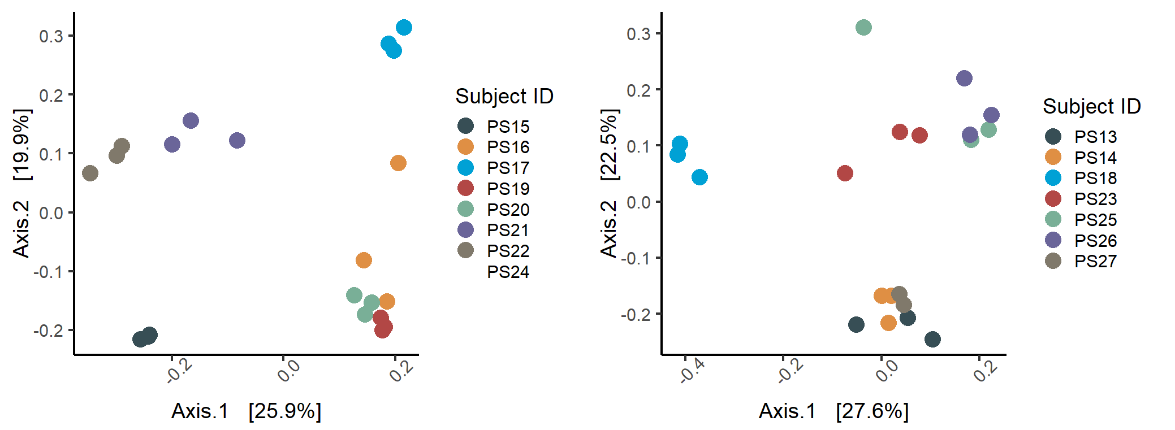


**Fig. S5: Diversity analyses of 16S rRNA sequencing of samples from human feeding trial.** A, B) Alpha diversity measurements (Shannon index) of samples pre (A) and post (B) treatment for each prebiotic group. C,D,E,F) Beta diversity ordination plots (PCoA) of bray-curtis dissimilarity matrix for samples in C) FOS responder, D) inulin responder, E) XOS responder and F) Non-responder groups. Each point represents a fecal sample and each color represents a different subject.
